# Tandem mass tag-based quantitative proteomic profiling identifies candidate serum biomarkers of drug-induced liver injury in humans

**DOI:** 10.1038/s41467-023-36858-6

**Published:** 2023-03-03

**Authors:** Kodihalli C. Ravindra, Vishal S. Vaidya, Zhenyu Wang, Joel D. Federspiel, Richard Virgen-Slane, Robert A. Everley, Jane I. Grove, Camilla Stephens, Mireia F. Ocana, Mercedes Robles-Díaz, M. Isabel Lucena, Raul J. Andrade, Edmond Atallah, Alexander L. Gerbes, Sabine Weber, Helena Cortez-Pinto, Andrew J. Fowell, Hyder Hussaini, Einar S. Bjornsson, Janisha Patel, Guido Stirnimann, Sumita Verma, Ahmed M. Elsharkawy, William J. H. Griffiths, Craig Hyde, James W. Dear, Guruprasad P. Aithal, Shashi K. Ramaiah

**Affiliations:** 1 Worldwide Research Development and Medical, Pfizer, USA; 2 NIHR Nottingham Biomedical Research Centre, Nottingham University Hospitals NHS Trust and the University of Nottingham, Nottingham, UK; 3 Translational Medical Sciences, School of Medicine, University of Nottingham, Nottingham, UK; 4 Servicios de Aparato Digestivo y Farmacología Clínica, Instituto de Investigación Biomédica de Málaga-IBIMA Plataforma Bionand, Hospital Universitario Virgen de la Victoria, Universidad de Málaga, Malaga, Spain; 5 Centro de Investigación Biomédica en Red de Enfermedades Hepáticas y Digestivas (CIBERehd), Madrid, Spain; 6 Department of Medicine II, University Hospital, LMU Munich, Munich Germany; 7 Clínica Universitária de Gastrenterologia, Faculdade de Medicina, Universidade de Lisboa, Lisbon, Portugal; 8 Department of Gastroenterology and Hepatology, Portsmouth Hospitals University NHS Trust, Portsmouth, UK; 9 Department of Gastroenterology and Hepatology, Royal Cornwall Hospitals NHS Trust, Cornwall, UK; 10 Department of Gastroenterology, Landspitali University Hospital Reykjavik, Reykjavík, Iceland; 11 Faculty of Medicine, University of Iceland, Reykjavík, Iceland; 12 University Hospital Southampton, Southampton, UK; 13 University Clinic for Visceral Surgery and Medicine, University Hospital Inselspital and University of Bern, Bern, Switzerland; 14 Brighton and Sussex Medical School and University Hospitals Sussex NHS Foundation Trust, Brighton, UK; 15 Liver Unit and NIHR Biomedical Research Centre, University Hospitals Birmingham NHS Foundation Trust, Queen Elizabeth Hospital, Birmingham, UK; 16 Department of Hepatology, Cambridge University Hospitals NHS Foundation Trust, Cambridge, UK; 17 Pharmacology, Therapeutics and Toxicology, Centre for Cardiovascular Science, University of Edinburgh, The Queen’s Medical Research Institute, Edinburgh, UK

**Keywords:** Diagnostic markers, Prognostic markers, Proteomics, Liver diseases

## Abstract

Diagnosis of drug-induced liver injury (DILI) and its distinction from other liver diseases are significant challenges in drug development and clinical practice. Here, we identify, confirm, and replicate the biomarker performance characteristics of candidate proteins in patients with DILI at onset (DO; *n* = 133) and follow-up (*n* = 120), acute non-DILI at onset (NDO; *n* = 63) and follow-up (*n* = 42), and healthy volunteers (HV; *n* = 104). Area under the receiver operating characteristic curve (AUC) for cytoplasmic aconitate hydratase, argininosuccinate synthase, carbamoylphosphate synthase, fumarylacetoacetase, fructose-1,6-bisphosphatase 1 (FBP1) across cohorts achieved near complete separation (range: 0.94–0.99) of DO and HV. In addition, we show that FBP1, alone or in combination with glutathione S-transferase A1 and leukocyte cell-derived chemotaxin 2, could potentially assist in clinical diagnosis by distinguishing NDO from DO (AUC range: 0.65–0.78), but further technical and clinical validation of these candidate biomarkers is needed.

## Abstract (web summary)

Diagnosis of rare, unpredictable, drug-induced liver injury (DILI) is a significant challenge for patients, clinicians, and drug development. Here, the authors discover, evaluate, and validate potential blood biomarkers to diagnose DILI and distinguish it from alternative causes of liver injury.

## Introduction

Drug-induced liver injury (DILI) is a major clinical problem associated with significant morbidity and mortality. Most cases of DILI recover after early detection of causative medication and its discontinuation. Persistence of DILI symptoms is associated with reduced quality of life^1^. Patients who present with acute DILI and concomitant jaundice have been found to have about 10% risk of mortality or need for liver transplantation^2^. A recent long-term follow-up of DILI patients found that progressive injury contributes to death in 7.6% of patients^3^. Moreover, DILI is one of the leading causes for termination of drug development programs and frequently the source of post-marketing regulatory actions^4,5^. Early detection and diagnosis of DILI is a major challenge as current biomarkers do not distinguish DILI from acute liver injury due to other etiologies. In clinical practice, elevation of biomarkers such as alanine aminotransferase (ALT), aspartate aminotransferase (AST), and alkaline phosphatase (ALP) are used as indicators of hepatocyte or biliary cell injury, along with total bilirubin (TBL) concentration that reflects liver dysfunction. However, raised levels of enzymes may also be found in cardiac and skeletal muscle diseases^6,7^. In addition, the widely accepted prognostic model, Hy’s law, is limited in predicting DILI outcome due to its poor specificity for acute liver failure^8–10^.

International collaborative efforts between Critical Path Institute’s Predictive Safety Testing Consortium (PSTC), Safer and Faster Evidence-Based Translation (SAFE-T) consortium, and Drug-Induced Liver Injury Network (DILIN) setup a guideline for identifying and qualifying candidate biomarkers^11^. They also reported total cytokeratin 18 (CK18), osteopontin (OPN), and macrophage colony-stimulating factor receptor (MCSFR) as potential prognostic DILI biomarkers and microRNA-122 (miR-122) and glutamate dehydrogenase (GLDH) as alternatives to ALT^12^. Nevertheless, biomarkers that can detect and diagnose DILI accurately remain elusive.

This study utilized mass spectrometry (MS) with higher-order multiplexing via an isobaric labeling strategy to simultaneously identify and quantify serum proteins in multiple cohorts as sensitive and specific biomarkers for early detection and diagnosis of DILI. We combined Tandem Mass Tag (TMT) based reporter methodology with MS instrumentation capable of providing quantitative accuracy using synchronous precursor MS3 analysis that eliminates interference. Then we developed a targeted MS assay to assess the performance characteristics of the selected candidate biomarkers in a second longitudinal confirmatory cohort. Finally, the performance characteristics of top biomarkers were tested in a third, multicenter, prospective cohort.

## Results

### Discovery proteomics

**Fig. 1:**
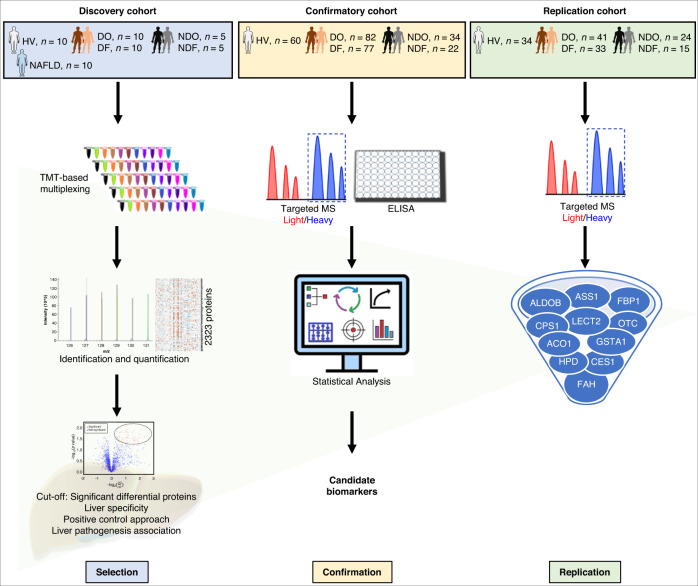
Schematic overview of the strategy for discovery, confirmation, and replication of DILI candidate biomarkers. Biomarkers cytoplasmic aconitate hydratase (ACO1), fructosebisphosphate aldolase B (ALDOB), argininosuccinate synthase (ASS1), liver carboxylesterase 1 (CES1), carbamoylphosphate synthase (CPS1), fructose-1,6-bisphosphatase 1 (FBP1), fumarylacetoacetase (FAH), glutathione S-transferase A1 (GSTA1), 4-hydroxyphenylpyruvate dioxygenase (HPD), leukocyte cell-derived chemotaxin-2 (LECT2), ornithine carbamoyltransferase (OTC) were identified using tandem mass tagging (TMT) mass spectrometry (MS) and subsequent ratio of light peptide/heavy isotopically labeled peptide or enzyme-linked immunosorbent assay (ELISA) in serum from healthy volunteers (HV), patients with nonalcoholic fatty liver disease (NAFLD), DILI or non-DILI, either at onset (DO or NDO, respectively) or at follow-up (DF or NDF).

During the discovery stage, 2323 proteins were identified in a cohort comprising patients with DILI, sampled at onset (DO; *n* = 10) and at follow-up (DF; *n* = 10), other acute liver injury (non-DILI), at onset (NDO; *n* = 5) and at follow-up (NDF; *n* = 5), chronic non-alcoholic fatty liver disease (NAFLD; *n* = 10), and healthy volunteers (HV; *n* = 10) (Fig. 1, Table 1, and Supplementary Table 1). Levels of the traditional biomarkers ALT, ALP and TBL all decreased in the follow-up visits (after 8–227 days) for 90% of DILI patients. Levels were also lower in chronic disease (NAFLD) compared to acute liver injury (DO and NDO).

**Table 1:** Demographics and clinical characteristics of participants in discovery, confirmatory, and replication cohorts

	*n*	Age, mean ± SD	Male/female (%)	Most frequent causative agents/conditions	ALT, IU/L median (IQR)	ALP, IU/L median (IQR)	TBL, mg/dL median (IQR)
**Discovery cohort**							
HV	10	57 ± 13	60/40		<45	<130	<1.2
DO	10	60 ± 17	60/40	Flucloxacillin (4), amoxicillin-clavulanate (2), atorvastatin (2)	166 (106–294)	272 (221–364)	2.4 (1–4.6)
DF	10	60 ± 17	60/40		32 (19–67)	130 (89–164)	0.8 (0.6–1.6)
*Mean days between DO and DF: 87*							
NDO	5	61 ± 15	20/80	Acute biliary obstruction (2), viral hepatitis, autoimmune hepatitis, other	241 (125–758)	324 (260–365)	2.9 (1.1–3.0)
NDF	5	61 ± 15	20/80		41 (35–51)	146 (89–198)	0.8 (0.5–1.0)
*Mean days between NDO and NDF: 51*							
NAFLD	10	58 ± 15	60/40		51 (42–61)	126 (84–163)	0.8 (0.5–1.0)
**Confirmatory cohort**							
HV	60	51 ± 13	32/68		<45	<130	<1.2
DO	82	54 ± 18	54/46	Amoxicillin-clavulanate (14), atorvastatin (7), ipilimumab/nivolumab (6), flucloxacillin (5), infliximab (4), nitrofurantoin (4), ibuprofen (4), metamizole (3)	339 (184–832)	229 (136–344)	3.6 (0.9–11)
DF	77	54 ± 17	53/47		46 (28–90)	96 (73–149)	0.9 (0.6–1.2)
*Mean days between DO and DF: 71*							
NDO	34	51 ± 20	38/62	Viral hepatitis (16), autoimmune hepatitis (10), other (8)	686 (282–1251)	237 (167–319)	10 (3.8–19)
NDF	22	56 ± 20	36/64		56 (29–214)	124 (92–183)	2.0 (0.6–10)
*Mean days between NDO and NDF: 47*							
**Replication cohort**							
HV	34	41 ± 17	35/65		<45	<130	<1.2
DO	41	56 ± 17	27/73	Flucloxacillin (8), atorvastatin (7), ipilimumab/nivolumab (3), herbal/dietary supplements (3), amoxicillin-clavulanate (2)	371 (146–781)	297 (141–410)	4.8 (1.2–14)
DF	33	57 ± 17	27/73		59 (30–141)	124 (105–168)	0.8 (0.6–2.4)
*Mean days between DO and DF: 63*							
NDO	24	53 ± 17	42/58	autoimmune hepatitis (12), viral hepatitis (8), other (4)	530 (245–998)	210 (172–316)	5.6 (2.1–21)
NDF	15	54 ± 16	40/60		128 (78–514)	202 (120–313)	2.3 (1.1–11)
*Mean days between NDO and NDF: 57*							

*ALP* alkaline phosphatase, *ALT* alanine aminotransferase, *DO* DILI onset, *DF* DILI follow-up, *HV* healthy volunteers, *IQR* interquartile range, *NAFLD* nonalcoholic fatty liver disease, *NDO* non-DILI onset, *NDF* non-DILI follow-up, *SD* standard deviation, *TBL* total bilirubin.

**Fig. 2:**
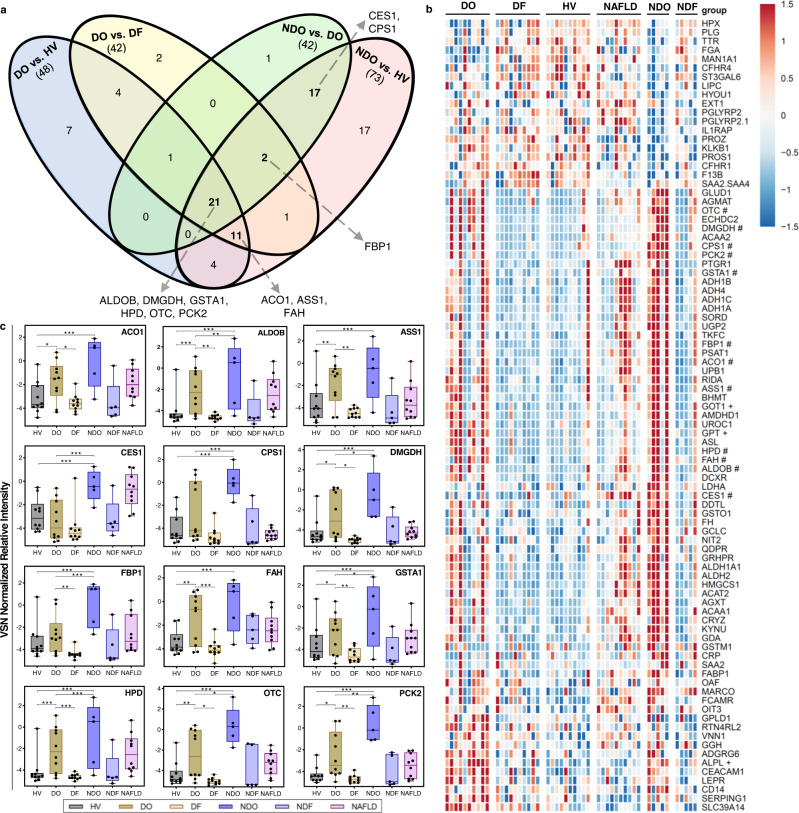
Discovery proteomics to identify candidate biomarkers. **a** Venn diagram showing statistically significant differentially expressed, liver-enriched proteins (p-value <0.1, two-sided and Benjamini-Hochberg-adjusted) between DILI onset (DO, *n* = 10) and healthy volunteer (HV, *n* = 10) samples, DO and DILI follow-up (DF, *n* = 10) samples, DO and non-DILI onset (NDO, *n* = 5), NDO and HV. The arrows indicate the liver-enriched candidate biomarkers selected from each group-wise comparison. **b** Heatmap of the 89-liver enriched, differentially expressed proteins (encoded by 88 genes) between all the 6 groups; + indicates the traditional biomarkers and # indicates selected candidate biomarkers. The variance stabilizing normalization (VSN) normalized expression data were converted to z-scores by row and represented with a color scheme ranging from -1.5 (blue), 0 (white), 1.5 (red), in 101 color step gradients. Row-wise, white represents values at the mean, with red and blue representing values of at least 1.5 standard deviations above or below the mean, respectively. **c** VSN normalized relative expression of candidate biomarkers identified from the analysis (two-sided and Benjamini-Hochberg-adjusted) shown in **a**, HV (*n* = 10), DO (*n* = 10), DF (*n* = 10), NDO (*n* = 5), NDF (*n* = 5) and NAFLD (*n* = 10). The center line in the box corresponds to the median, the box represents the first and third quartiles, and the whiskers represent the minimum and maximum observed values. *P < 0.1; **P < 0.01; ***P < 0.001. Source data are provided as a Source Data file.

Out of the 2323 proteins, we first identified proteins where the relative expression levels significantly differed between the experimental groups using the following pairwise comparisons: DO versus HV, DO versus DF, NDO versus DO, and NDO versus HV. The differentially expressed proteins were filtered for ‘liver enriched genes’^13^ to identify candidates with liver-specific expression. The comparative analysis identified 48 significant proteins between DO and HV, 42 proteins between DO and DF, 42 proteins between NDO and DO, and 73 between NDO and HV. As shown in the Venn diagram (Fig. 2a) a total of 89 proteins (~4% of the proteins from the total proteome) (Fig. 2b), showed significant differential expression in respective pairwise comparisons. We first focused on a subset of 51 proteins (highlighted in bold in Fig. 2a) that were present in at least two comparisons. Out of these 51 proteins, we found that 21 proteins were shared among all the groups (including traditional markers, ALT and AST) and 2 were shared between DO, NDO, and HV. Seventeen proteins were able to differentiate NDO from DO and HV. Only 11 proteins (including ALP) differentiated DO from HV and DF (Fig. 2a). These 51 proteins were then ranked and short-listed for further investigations.

### Selection of candidate biomarkers

The 51 proteins were ranked based on (1) differential expression, (2) liver specificity, and (3) mechanistic relevance to liver biology; and top candidate proteins were short-listed (Fig. 2a). These included: cytoplasmic aconitate hydratase (ACO1); argininosuccinate synthase (ASS1); fumarylacetoacetase (FAH); carbamoylphosphate synthase (CPS1); fructosebisphosphate aldolase B (ALDOB); 4-hydroxyphenylpyruvate dioxygenase (HPD); ornithine carbamoyltransferase (OTC); dimethylglycine dehydrogenase (DMGDH); glutathione S-transferase A1 (GSTA1); fructose-1,6-bisphosphatase 1 (FBP1); mitochondrial phosphoenolpyruvate carboxykinase 2 (PCK2); liver carboxylesterase 1 (CES1). These candidate proteins were taken forward to quantitatively assess their performance as potential biomarkers of DILI. In addition, we selected leukocyte cell-derived chemotaxin 2 (LECT2), a hepatokine highly expressed in the liver that is involved in liver injury and liver regeneration^12^. Although LECT2 did not meet our significance threshold in the discovery analysis, it was elevated in DO compared to NDO (Supplementary Fig. 1).

Levels of each biomarker were compared across the 6 discovery cohort groups (Figs. 1, 2c and Supplementary Fig. 1). The serum levels of CPS1, HPD, FBP1, CES1 (*P* < 0.001), ALDOB, PCK2 (*P* < 0.01), DMDGH, GSTA1, and OTC (*P* < 0.1), were all lower in DO patients than in NDO in contrast to LECT2. The expression of all proteins was significantly different between NDO vs HV (*P* < 0.001) and the median levels of all except CES1 and PCK2 were lower in NAFLD than DO.

### Performance characteristics of biomarkers in differentiating DILI from HV

**Fig. 3:**
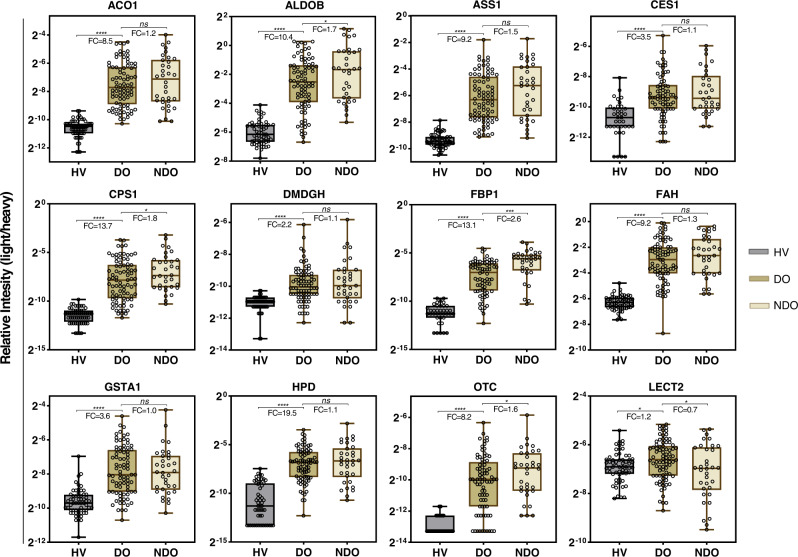
Performance of candidate biomarkers in confirmatory cohort. Relative intensity levels of selected candidate biomarkers differentially present in DILI onset (DO, *n* = 82) compared with healthy volunteers (HV, *n* = 60) and non-DILI onset (NDO, *n* = 34). The center line in the box corresponds to the median, the box represents the first and third quartiles, and the whiskers represent the minimum and maximum observed values. *P* (*t* test, two-sided, no adjustment) and fold change (FC) values are presented in the boxes (*P < 0.05; **P < 0.01; ***P < 0.001; ****P < 0.0001; *ns* = not significant). Source data are provided as a Source Data file.

The performance characteristics of these candidate protein biomarkers were next determined in a confirmatory cohort (Fig. 1) that included samples from DO (*n* = 82), DF (*n* = 77), NDO (*n* = 34), NDF (*n* = 22) and HV (*n* = 60) (Fig. 3 and Supplementary Fig. 2). All proteins except PCK2 were significantly increased in DO by 1.2-fold to 19.5-fold compared to HV (*P* < 0.0001) and all proteins, except for LECT2, were decreased in DF (Supplementary Fig. 2, 3). We next evaluated the ability of each protein to detect liver injury (DO versus HV) by Area Under the Curve (AUC) Receiver Operating Characteristic (ROC) analysis (Supplementary Table 2). The routinely used biomarkers ALT, AST, ALP, and TBL all had AUCs 0.92–1.0, whereas GLDH and CK18, previously identified as putative DILI biomarkers^12^, had AUCs of 0.86 and 0.96, respectively. The candidate biomarkers, ACO1, ASS1, FAH, FBP1, and CPS1 (AUCs: 0.99, 0.98, 0.98, 0.96 and 0.96, respectively) achieved near-complete separation between patients with liver damage and healthy subjects (Supplementary Table 2). ALDOB, HPD, OTC, GSTA1, DMGDH, and CES1 (AUCs: 0.94, 0.94, 0.92. 0.87, 0.86 and 0.80, respectively) showed high to moderate separation between the groups. This indicates that these proteins are the most accurate candidate biomarkers for the detection of DILI, whereas LECT2 (AUC 0.61) and PCK2 (AUC 0.56) were not able to separate DO from HV (Supplementary Table 2).

We next replicated these findings by determining the AUC-ROC of the candidate biomarkers in samples from a third cohort (replication cohort) consisting of patients with DO (*n* = 41), DF (*n* = 33), NDO (*n* = 24), NDF (*n* = 15) and HV (*n* = 34) (Fig. 1, Table 1, Supplementary Table 1). Similar results were obtained for the leading biomarkers which included ACO1, ASS1, FAH, FBP1 and CPS1 (AUCs: 0.98, 0.97, 0.99, 0.94 and 0.95, respectively; Supplementary Table 3).

Multivariate models to differentiate DILI from HV built using logistic regression (AUC = 0.95) and random forest (RF) approaches (AUC = 0.99) with the confirmatory cohort dataset were compared (Supplementary Fig. 4a). The importance score of each biomarker was then calculated for the predictive RF model including all 12 candidate biomarkers (Supplementary Fig. 4b). Candidate biomarkers ACO1, FAH, ASS1, FBP1, ALDOB, and CPS1 formed a high-score group (scoring 90, 90, 86, 76, 59, and 54 out of 100, respectively) and were subsequently used to build 4 prediction models containing different combinations of these biomarkers (Supplementary Fig. 4c). The replication cohort was used as an independent validation dataset to test the AUCs (Supplementary Fig. 4d). Interestingly, all four models had AUCs > 0.97 (Supplementary Fig. 4d), comparable to the AUCs of individual proteins (Supplementary Tables 2 and 3).

### Performance characteristics of biomarkers in distinguishing DO from NDO

All candidate protein biomarkers were tested in a confirmatory cohort to verify the performance in distinguishing DILI from acute non-drug related liver injury. When comparing DO versus NDO, only serum ALDOB (*P* < 0.05, fold change (FC) FC = 1.7), CPS1 (*P* < 0.03, FC = 1.8), LECT2 (*P* < 0.03, FC = 0.7), OTC (*P* < 0.04, FC = 1.6), and FBP1 (*P* < 0.001, FC = 2.6) levels were significantly altered in NDO patients compared to DO (Fig. 3). All other markers did not show any significant difference between the two groups, which was consistent with the findings in the discovery cohort.

We next evaluated each candidate biomarker’s ability to differentiate DO from NDO by comparing to AUCs of traditional biomarkers and previously identified biomarkers, (Supplementary Table 2). ALT, AST, ALP, TBL had AUCs 0.53–0.65, whereas previously identified biomarkers such as GLDH and CK18 had AUCs of 0.48 and 0.66, respectively. FBP1 had the highest AUC (0.75), followed by PCK2 (0.63), LECT2 (0.62), CPS1 (0.61), OTC (0.61), and ALDOB (0.60). The AUCs for remaining candidate biomarkers ranged from 0.47 to 0.59 (Supplementary Table 2).

An independent replication cohort was used to test the performance of biomarkers in distinguishing DILI from acute non-drug related liver injury and a consistent trend was observed between confirmatory and replication cohorts (Supplementary Table 3).

Multivariate models were constructed to identify the distinguishability of DILI from acute non-drug related liver injury. Logistic regression (AUC = 0.65) and RF (AUC = 0.68) models (Supplementary Fig. 5a) were trained, tested, and validated using the replication cohort data as an independent validation dataset. Using a logistic regression approach, FBP1, GSTA1, LECT2, and CES1 (importance scores: 95, 58, 51, and 48, respectively) in the high score group of biomarkers were used to build the models with a combination of two or three biomarkers (Supplementary Fig. 5b). With the RF approach, FBP1, LECT2, and CPS1 (importance scores: 100, 42, and 12, respectively) were used to build the model (Supplementary Fig. 5c, d).

Out of the five multivariate models, RF models (FBP1 + LECT2 and FBP1 + LECT2 + CPS1) tended to have a higher AUC (Table 2) for the confirmatory cohort dataset with lower AUC (0.64 and 0.61, respectively) for the replication cohort dataset, which suggests that a potential over-fit and cross validation was used in the model fitting. The differences between confirmatory cohort AUCs (0.75–0.78) and replication cohort AUCs (0.64–0.69) for the logistic regression models were smaller.

**Table 2:** Assessment of logistic regression and random forest biomarker models in the confirmatory and replication cohorts

Method	Biomarkers/Models	AUC of confirmatory cohort between NDO vs. DO	AUC of replication cohort between NDO vs. DO
Logistic regression	FBP1 + GSTA1	0.75	0.69
	FBP1 + GSTA1 + LECT2	0.78	0.68
	FBP1 + CES1 + LECT2	0.78	0.64
Random forest	FBP1 + LECT2	1.00	0.64
	FBP1 + LECT2 + CPS1	1.00	0.61

*AUC* area under the receiver operating characteristic curve, *NDO* non-DILI patients at onset, *DO* DILI patients at onset. See also Supplementary Fig. 5.

**Fig. 4:**
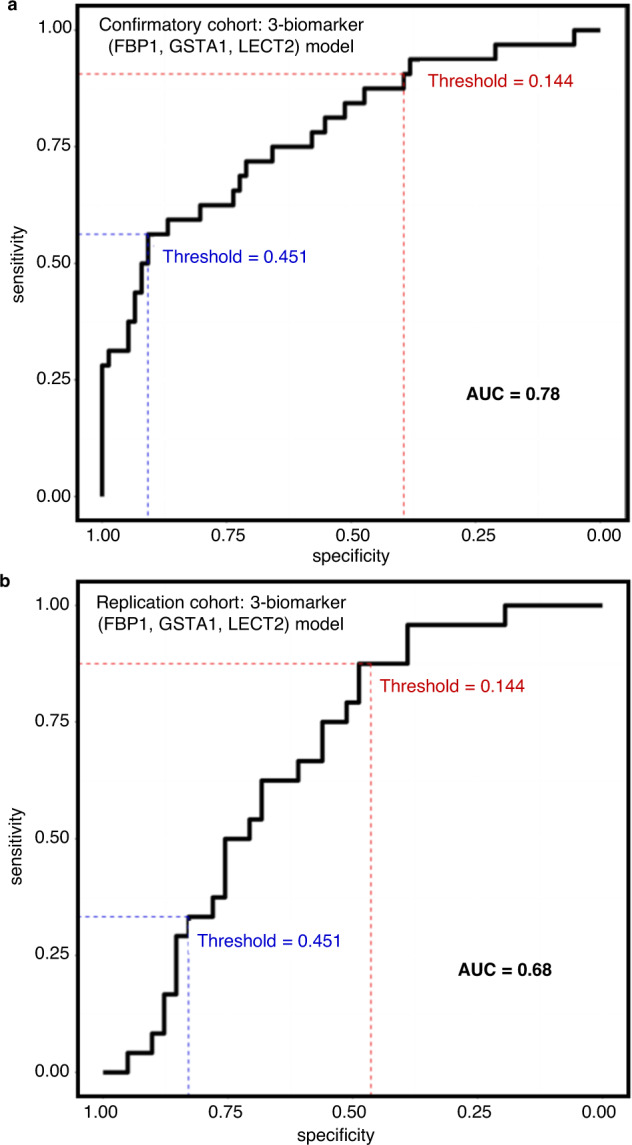
AUC-ROC analysis of multivariate biomarker models. AUC-ROC shown for FBP1 + GSTA1 + LECT2 biomarker model in non-DILI patients at onset (NDO) versus DILI patients at onset (DO) in the confirmatory cohort (*n* = 116) (**a**) and the replication cohort (*n* = 65) (**b**).

To compare the performance of multivariate models (where NDO = 1, DO = 0), the specificity was set to ≥0.90, and sensitivity then compared within the confirmatory cohort (Table 3). The threshold was determined by maximizing the sensitivity given specificity ≥ 0.90. The logistic regressions using 2- and 3-biomarker models had similar specificities and exhibited strong predictability, as reflected by the AUC (Fig. 4a, b, Table 3) when comparing NDO to DO in the confirmatory cohort. Interestingly in the replication cohort, the FBP1 + GSTA1 + LECT2 model demonstrated a similar performance, but slightly lower specificity was observed for other models. Similarly, when setting the sensitivity ≥ 0.90 and specificity was compared, the FBP1 + GSTA1 + LECT2 model displayed the best performance compared to the other models suggesting that this model has a higher specificity for identifying patients with DILI than any of the other models. However, the multivariate model’s predictability was not enhanced when we included the traditional biomarkers (Supplementary Tables 4, 5). In summary, FBP1 might be a promising standalone biomarker in differentiating DILI from other types of acute liver injury. This biomarker candidate may also provide mechanistic insights for DILI as it was uniquely associated with cluster 15, also known as hep 6 interzonal region, in a transcriptional map of human liver acinus^14^ (Supplementary Fig. 6).

**Table 3:** Comparative assessment of candidate biomarker multivariate models at a fixed specificity and sensitivity for the diagnosis of liver injury

Metric	Model	Threshold	Confirmatory cohort						Replication cohort					
**Specificity** ≥ **0.90**			Specificity	Sensitivity	TN	TP	FN	FP	Specificity	Sensitivity	TN	TP	FN	FP
Logistic regression	FBP1 + GSTA1	0.50	0.92	0.31	70	10	22	6	0.90	0.13	37	3	21	4
	FBP1 + GSTA1 + LECT2	0.45	0.91	0.56	69	18	14	7	0.83	0.33	34	8	16	7
	FBP1 + CES1 + LECT2	0.52	0.91	0.47	69	15	17	7	0.85	0.21	35	5	19	6
Random forest	FBP1 + LECT2	0.46	1.00	1.00	76	32	0	0	0.83	0.42	34	10	14	7
	FBP1 + LECT2 + CPS1	0.46	1.00	1.00	76	32	0	0	0.76	0.46	31	11	13	10
**Sensitivity** ≥ **0.90**														
Logistic regression	FBP1 + GSTA1	0.14	0.36	0.91	27	29	3	49	0.29	0.96	12	23	1	29
	FBP1 + GSTA1 + LECT2	0.14	0.39	0.91	30	29	3	46	0.46	0.88	19	21	3	22
	FBP1 + CES1 + LECT2	0.16	0.41	0.91	31	29	3	45	0.46	0.79	19	19	5	22
Random forest	FBP1 + LECT2	0.46	1.00	1.00	76	32	0	0	0.83	0.42	34	10	14	7
	FBP1 + LECT2 + CPS1	0.46	1.00	1.00	76	32	0	0	0.76	0.46	31	11	13	10

Each model compared onset non-DILI (NDO) cases versus DILI cases (DO) and was trained using the confirmatory cohort and validated using the replication cohort.

*TP* true positive, *TN* true negative, *FP* false positive, *FN* false negative.

### Spearman rank correlation analysis between candidate biomarkers and ALT

**Fig. 5:**
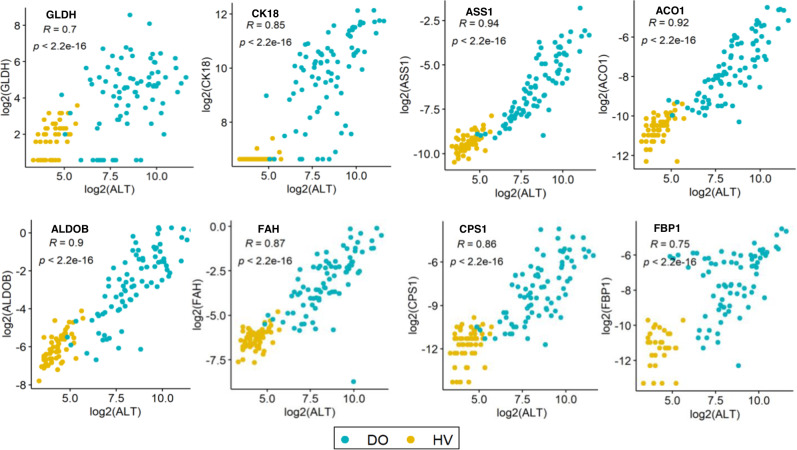
Correlation between biomarkers and ALT. Spearman’s r correlation coefficient (R) (test R = 0 or not, two-sided, no adjustment) was calculated between ALT and either previously identified biomarkers (glutamate dehydrogenase (GLDH) and cytokeratin-18 (CK18)) or candidate biomarkers. Circles indicate HV (*n* = 60) and DO (*n* = 82) samples from the confirmatory cohort. Values are log2 transformed and *p* values are shown. Source data are provided as a Source Data file.

To evaluate biomarker specificity for liver injury we assessed Spearman’s r correlation coefficient between levels of each putative biomarker (including previously identified biomarkers, GLDH and CK18 and our candidate biomarkers) and ALT activity, in HV and DO cases of the confirmatory cohort (Fig. 5). GLDH (*R* = 0.70) and CK18 (*R* = 0.85) showed strong correlation with ALT. The candidate biomarkers ASS1 (*R* = 0.94), ACO1 (*R* = 0.92), ALDOB (*R* = 0.90), FAH (*R* = 0.87), CPS1 (*R* = 0.86), HPD (*R* = 0.82), OTC (*R* = 0.81) and FBP1 (*R* = 0.75) demonstrated a stronger correlation with ALT than GLDH or CK18 (Fig. 5 and Supplementary Fig. 7). All other biomarkers correlated poorly with ALT in the confirmatory cohort.

## Discussion

There are significant gaps in the current tools available for the diagnosis of DILI, which mainly relies on establishing a temporal relationship between drug exposure and liver injury and performing extensive tests to exclude alternative etiologies^15^. A recent systematic review highlighted the urgent need for biomarkers that distinguish DILI from acute liver injury due to alternative etiologies^16^. We addressed this issue by not only investigating the ability of biomarkers to ‘detect DILI’, but also testing their performance characteristics in ‘distinguishing DILI from other causes of acute liver injury’. In the present large, multicenter case-control study, involving 133 patients with DILI and 104 healthy controls, we found candidate protein biomarkers ACO1, ASS1, FAH, FBP1, and CPS1 that were able to detect DILI (from healthy controls) with high accuracy (AUC > 0.90). In addition, FBP1 alone or in combination with GSTA1 and LECT2 was able to distinguish acute liver injury due to non-drug etiology from DILI with AUCs of 0.75 and 0.78 in the confirmatory cohort and 0.65 and 0.68, respectively in the replication cohort. Combining multiple biomarkers has potential for assisting in the clinical diagnosis of DILI.

Our study design reflects the clinical context by recruiting patients presenting (or referred) with manifestations of acute liver injury as evidenced by elevation of liver enzymes and/or bilirubin. Our strategy is similar to what has been previously described as ‘screen-and-confirm’ approach^17^, referring to use of conventional biomarkers such as liver enzymes to ‘screen’ for initial signals and then using new biomarkers for in-depth investigations to ‘confirm’ DILI. The biomarker panel including FBP1, GSTA1, and LECT2 demonstrated the best performance characteristics as a diagnostic test. Considering the potential clinical application of the biomarker, we have identified multiple cut-off values where this biomarker panel has high sensitivity (90%) or high specificity (90%). These cut-offs can be used to rule-in or rule-out DILI depending on the clinical context of use. We can illustrate the application of the putative biomarker panel in the following three clinical scenarios. First, Breu et al.^18^ found that 11.3% of patients with ≥1000 IU/L ALT or AST (defined as ‘acute liver injury’ by the authors) had DILI. Therefore, using a cut-off value of 0.45 for a positive biomarker panel (FBP1 + GSTA1 + LECT2) test (Table 2, Fig. 4a), the post-test probability for the diagnosis of DILI would be 21%. Similarly, Donaghy et al.^19^ estimated that in patients presenting with ‘hepatic jaundice’ (where biliary obstruction is ruled out by imaging), the pre-test probability of DILI would be 15%; in this context, our positive biomarker panel test would hence result in a post-test probability of 27%. Third, using the Veterans Health Administration corporate data warehouse, a national electronic health record repository of clinical and administrative data, Suzuki A et al.^20^ identified patients with one of two indicators of acute liver injury (ALT ≥ 5x ULN, or ALP ≥ 2x ULN) within 90 days from amoxicillin-clavulanate initiation. They found the pre-test probability of DILI in their cohort to be 35%; if our biomarker panel test is positive in this situation, then the post-test probability would be 53%.

**Fig. 6:**
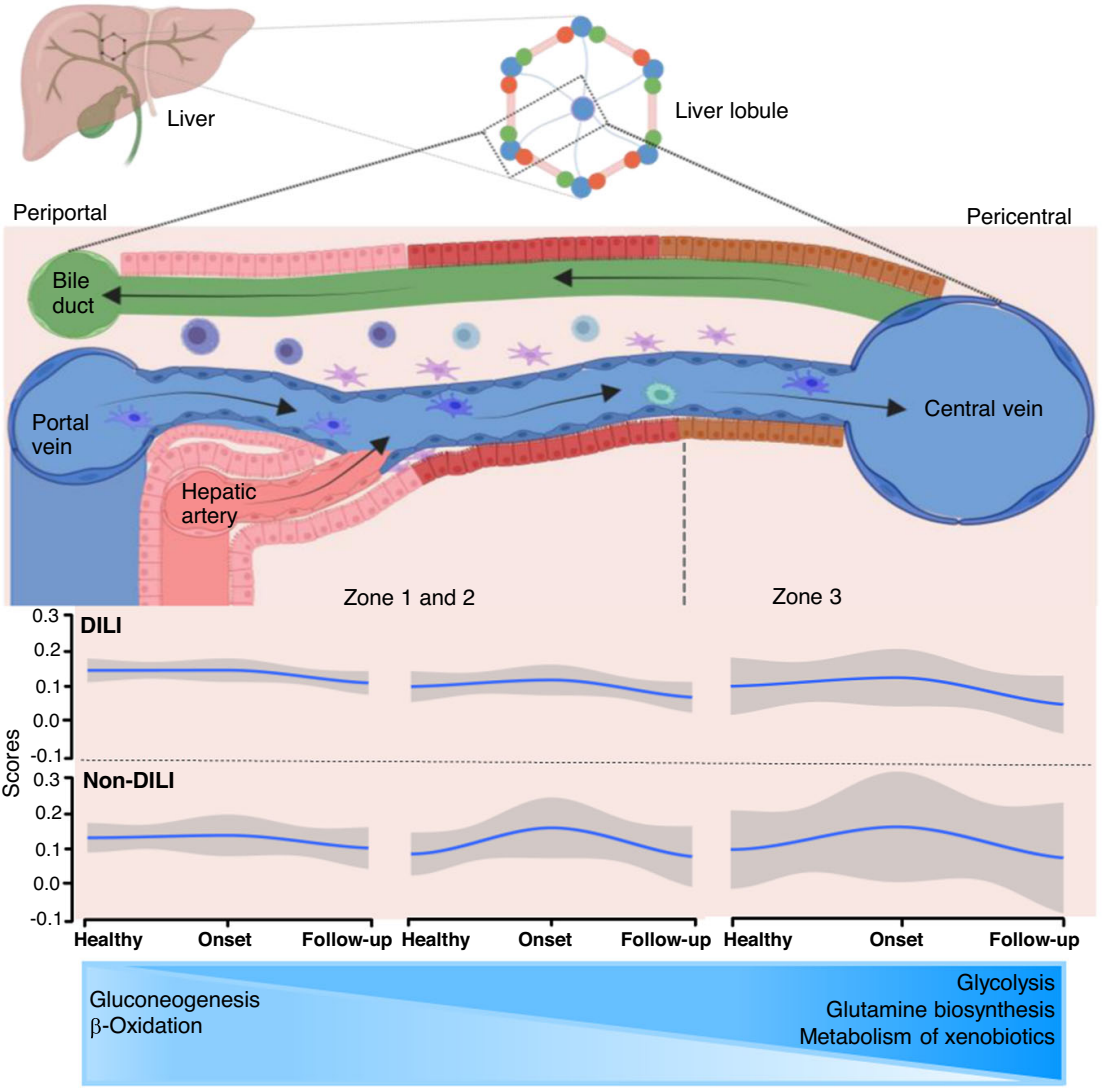
Visualization of liver pathway signatures. Visualization of apparent differences in liver zone pathway signatures among NDO, DO, and HV, using single sample gene set enrichment analysis of the discovery proteomics dataset. Pathway enrichment from differentially expressed genes between NDO (*n* = 5) and HV (*n* = 10), and NDO and DO (*n* = 10) shown in Supplementary Fig. 6. Pathway scores are calculated from expression data for each gene set to allow side-by-side visualization. Healthy volunteers (HV) scores were combined with DILI onset and follow-up (DO and DF (*n* = 10)) or with non-DILI onset and follow-up (NDO and NDF (*n* = 5)) to simulate transition through healthy, onset, and follow-up in either DILI or non-DILI disease. The structure of individual lobules in the liver is represented having parenchymal cells (hepatocytes) and multiple non-parenchymal cells aligned between a capillary network and a central vein and liver interzonal signature expression plotted below the lobule zones. Smooth lines were generated by loess method, gray indicates standard deviation. Zonal expression levels of differentially expressed proteins which are involved in gluconeogenesis, β-oxidation, glycolysis, glutamate biosynthesis, and xenobiotics metabolism is illustrated below the graphs. Image was made with BioRender (# FR23R67B3Z). Source data are provided as a Source Data file.

Our study found significantly higher expression of FBP1 in acute non-DILI patients compared to DILI, with a good performance characteristic for the biomarker in distinguishing the two etiologies (Table 2 and Supplementary Table 2). Multivariate models with combinations of GSTA1, LECT2, CES1 and CPS1, demonstrated that the performance characteristics of FBP1 in separating DILI from liver injury due to other etiologies improved further (Table 3). FBP1 is predominantly expressed in the liver and is responsible for the hydrolysis of fructose 1,6-bisphosphate to fructose 6-phosphate in the gluconeogenesis pathway. As previously reported, elevation of FBP1 suggests that increased glucose metabolism protects the liver from injury induced by hepatic apoptosis^21^. Serum levels of FBP1 have been shown to be of prognostic value in acute liver failure associated with 30-day survival in end-stage liver disease^22^. FBP1 has been shown to be uniquely associated with hep 6 interzonal region of human liver acinus^14^ (Fig. 6 and Supplementary Fig. 6). Overall, 55% of hepatocyte proteins are zonated^23^, although further investigations are needed to understand why markers unique to the interzonal region may serve to differentiate DO from NDO. We also linked the gene signatures derived from spatial transcriptomics to differentially expressed liver enriched proteins, which mapped GSTA1, OTC, ALDOB and FBP1 to mid-lobule hepatocytes.

Within the confirmatory cohort LECT2 was the only candidate biomarker that was significantly increased in DO compared to NDO (Fig. 3). The hepatokine LECT2 enhances lipopolysaccharide (LPS) stimulated activation of tissue macrophages contributing to the development of inflammation^24^. Interestingly, a non-toxic dose of trovafloxacillin coupled with LPS-induced inflammation caused acute liver injury in rodent models^25^. LECT2, considered to be a biomarker of regeneration, has been shown to be a prognostic indicator of acute liver failure^26^, whereas GSTA1, a phase II detoxification enzyme primarily enriched in the liver, is considered a marker of hepatocyte injury in vitro and an early indicator of liver injury in mice^27,28^.

Some of the candidate biomarkers identified in this study have been previously reported in animal models of acetaminophen-induced liver injury^29–33^, and human DILI^12,29–31,34^ and described as having roles in liver injury or recovery^35–37^. However, here we have comprehensively evaluated the biomarker performance characteristics of these proteins in comparison with other established or proposed biomarkers.

Although serum ALT and GLDH elevations are accepted to be relatively liver-specific, ALT can be associated with muscle injury, and elevated levels of GLDH have been observed without hepatotoxicity^6,37,38^; therefore a biomarker with greater liver specificity could be advantageous for the detection of DILI in specific clinical settings. ALDOB, CPS1, and OTC are highly liver-specific and performed well against GLDH. ALDOB, a component of the glycolytic pathway, was significantly higher in DO compared with HV and showed a strong correlation with ALT activity (*R* = 0.9) (Fig. 5). This is consistent with observed troglitazone-induced hepatotoxicity in humans associated with the appearance of autoantibodies to ALDOB, and the correlation between ALDOB elevation and the extent of liver dysfunction^34,39^. In addition, CPS1 and OTC, which are mitochondrial urea cycle enzymes, were elevated in the DO patients and correlated well with ALT activity (*R* = 0.86 and 0.81). This aligns with reports that CPS1 is released into the bloodstream during acetaminophen-related acute liver injury in mice as well as humans and could potentially indicate prognosis^30,40^. Furthermore, CPS1 has been proposed as an early indicator of recovery from DILI due to its short serum half-life, whereas high ALT levels persist longer in the blood^30,41^. OTC is primarily expressed in hepatocytes and is released into the blood in rat liver dysfunction models^42^. It has been proposed to be an indicator of hepatotoxicity both in clinical and drug development settings^43^.

In the current study, the liver enriched proteins ACO1, ASS1, and FAH were elevated in DILI patients compared with HV and strongly correlated with ALT activity (*R* = 0.87–0.94). Interestingly, FAH and ASS1 were previously identified as promising specific biomarkers for liver injury using a proteomics approach^31,34^. The liver enzyme ASS1 has also been suggested as a specific indicator of liver injury in mouse models; it is elevated in acetaminophen overdose patients and can be detected in humans with moderate liver injury^29,31^. Nevertheless, additional focused mechanistic studies are needed to determine the roles and significance of all these candidate biomarkers.

The current study has several strengths. First, it was embedded within the clinical pathway followed by patients with DILI, which means that the biomarkers were evaluated in the context of their use. In addition, both DILI cases and acute non-DILI controls were thoroughly investigated with causality of DILI systematically assessed using the Roussel Uclaf Causality Assessment Method^44^ and etiology of liver injury was consistently adjudicated by an expert panel. Second, a large-scale systematic and comprehensive proteomic analysis by TMT-based isobaric tagging methodology was undertaken using DILI patients as well as several control groups. Most previous studies have not included acute non-DILI groups to test the ability of biomarkers to distinguish DILI from alternative acute non-DILI cases^16^. However, a previous smaller study conducted metabolomics profiling of 10 patients with DILI and compared it with chronic liver disease subgroups and hepatocellular cancer patients^45^, while another study^46^ compared metabolomic changes between 13 DILI patients, 12 with autoimmune hepatitis and 24 with viral hepatitis. These studies showed that metabolic markers have the potential to discriminate and improve the clinical diagnosis of DILI. At present, diagnosis of DILI is reliant on a compatible temporal relationship between drug exposure and liver injury and extensive investigations to exclude alternative explanations with no specific test done to support or rule out DILI diagnosis. Therefore, the panel that we have evaluated and validated has the potential to fill this gap.

This study has some limitations. Although DILI cases and acute non-DILI control groups were all enrolled using a single protocol, with pre-defined liver biochemistry thresholds, significant variability in characteristics persisted between these groups and within the groups (Supplementary Table 1). Individual drugs are also associated with DILI of different phenotypes, but the number of cases in the current study was insufficient for subgroup analyses among different biochemical patterns of injury or drug types. However, the design of this study is based on the premise that common, or a particular combination of mechanisms underpin DILI resulting from a broad group of drugs. Although ‘proximal’ steps (upstream events) in the development of DILI are likely to be drugs/class specific, ‘distal’ steps (downstream events) are likely to be shared by DILI due to multiple drugs, so may have common biomarkers. Similarly, the acute non-DILI control group was formed by acute liver injury due to a wide range of etiologies. Since we followed strict consecutive recruitment of available cases to maximize recruitment, the DILI and non-DILI cohorts could not be matched for age, sex, BMI. Furthermore, this resulted in differences in liver profile values between the DILI and acute non-DILI control group, with the latter mostly presenting higher values in all three cohorts. However, this reflects the case composition, since viral hepatitis and autoimmune hepatitis that comprised a large portion of the acute non-DILI liver injury group, are generally associated with higher liver profile values. Further refinement requires a larger cohort to enable analyses using specific acute non-DILI subtypes and DILI due to specific drugs. Enrollment is underway to address these limitations and to strengthen the findings reported in this study^47^.

Costs and limited access to new tests can be a barrier in the translation to clinical practice. The development and implementation of MS-based biomarker measurements in diagnostic or healthcare facilities would require substantial investments and expertise. Alternatively, enzyme-linked immunosorbent assay-based quantification is feasible with acceptable costs but needs development for routine use. Future integration of metabolic and/or genetic biomarkers from other studies may also further enhance this panel.

In conclusion, we identified candidate protein biomarkers that are able to detect DILI and distinguish it from acute non-drug related liver injuries in a prospective cohort of patients with manifestations of acute liver injury presenting to a secondary care hospital setting. If the diagnostic and prognostic performance of these biomarkers is validated in an independent large case-control study, could pave the way for a step change in clinical practice as well as in monitoring for DILI in clinical trials.

## Methods

### Participants

All study participants provided written informed consent. Studies complied with the Declaration of Helsinki, Good Clinical Practice (Directive 2001/20/EC) and general data protection regulations (EU) 2016/679. Suspected DILI cases were consecutively recruited at centers across Europe between April 2016 and July 2021: UK, Spain, Germany, Iceland, Portugal; approved by: Yorkshire and the Humber - Leeds East Research Ethics Committee (Ref15./YH/0294); Biomedical Investigation Ethics Committee of Andalucia (Ref: AND-HEP-2015-01); Ethical Commission of Ludwig Maximilian University of Munich (Project 85-16); Bioethics Committee Iceland (Ref: 15–104-V1); Ethics Comission of Centro Académico Médico de Lisboa (Ref: 126/15) and National Data Protection Comission Portugal (Authorization 479/2016), respectively. Standard clinical investigations including imaging were performed and cases were followed until liver profile normalization where possible.

Causality assessment was performed^48^ and the cases were reviewed by an expert panel of at least three experienced clinical hepatologists or clinical pharmacologists from three different European academic centres^47^. The panel adjudicated episodes as DILI or alternative diseases termed ‘acute non-DILI. They excluded patients where the investigations were inconclusive or when disagreement of probable diagnosis occurred within the panel. Both DILI and non-DILI groups met the same biochemical criteria as defined previously^48^, having serum ALT ≥ 5x ULN or ALT ≥ 3x ULN plus TBL ≥ 2x ULN or ALP ≥ 2x ULN with accompanying elevations of gamma-glutamyl transferase. DILI was diagnosed based on the presence of a compatible temporal sequence between drug intake and detection of liver injury as well as test results to exclude alternative conditions, which included, but were not limited to, viral serology, viral load, imaging tests, presence of autoantibodies, immunoglobulin G values and biopsy findings (when available). Table 1 and Supplementary Table 1 show clinical and demographic data, the primary causative drug implicated in DILI and etiology of acute non-DILI controls. Cases of acetaminophen overdose were not excluded and were eligible as an acute control, although none were recruited during the period of this study. The ‘onset’ samples for both DILI cases (DO) and acute non-DILI controls (NDO) were collected at the time of enrollment into the study. A second sample termed ‘follow-up’ was collected from both DILI (DF) and acute non-DILI control (NDF) group patients (median follow-up of 35 days after the enrollment). This was to explore the trajectory of biomarkers in relation to the course of the liver injury. Patients who had liver enzymes below ULN at the follow-up visit were considered to have ‘complete recovery’. Those patients where liver enzymes decreased at follow-up but remained above ULN were termed ‘partial recovery’ (Supplementary Table 6). Where there was no follow-up visit, levels recorded in clinical notes were used to define recovery type.

HV and patients with biopsy-proven chronic liver disease (NAFLD) were enrolled as additional control groups. These controls were sex and age matched to the DILI cases and were recruited in parallel in Nottingham for research approved by East Midlands Nottingham 2 research ethics committee (Ref: GM0102010). HV had no diagnosis of liver disease and for those included in the discovery cohort, absence of fatty liver was confirmed using controlled attenuation parameter of transient elastography or transabdominal ultrasound examination. Subsequent cohorts were not screened for fatty liver but had no diagnosis of liver disease. Serum samples obtained were stored at −80 °C until analysis. Both sexes were included in the study design. Sex data was collected based on self-reporting. Proportions in sub-groups are reported, but sex-based analyses could not be performed due to the small cohort size.

### Discovery experiment design

Global proteomics analysis involved Tandem Mass Tag (TMT) isobaric labeling of peptides for the identification and relative quantitation of proteins by multiplexing the 50 discovery cohort participants (Fig. 1) using 11-plex TMT reagents (randomized into 5 sets). The samples were analyzed using a Thermo Orbitrap Fusion MS. A list of biomarker targets was compiled using statistical significance between DO and HV, DO and DF, DO and NDO, NDO and HV (Fig. 2b).

### Discovery proteomics workflow

Immunoaffinity depletion: serum samples were depleted with a dual immune-affinity approach employing Seppro IgY14 (LC10) and Supermix (LC5) columns (Sigma) according to the manufacturer’s instructions. 100 μL of serum was diluted fivefold into 1X Seppro Dilution buffer and filtered using 0.45 μM spin filters at 4 °C for 10 min at 9000 g. An Agilent 1200 HPLC system was used for automated control of the dual depletion setup. Immunoglobulin (IgY14) and Supermix columns were connected in tandem through a column selection valve to allow for automated selection of the flow path. The diluted and clarified serum samples were injected onto the IgY14 column with the column selection valve set to flow through both columns in tandem, while running Buffer A (1X Seppro Dilution Buffer, Sigma) at 0.5 mL/min. The flow-through protein fraction was collected into a chilled (4 °C) autosampler using timed collection over four 3 mL fractions. Upon collection of the flow-through peak, the column selection valve was switched such that flow was directed solely through the IgY14 column, and 100% Buffer B (1X Seppro Stripping Buffer, Sigma) flowed at 2 mL per minute. Upon complete elution of the IgY14 bound fraction, proteins bound to the supermix column were eluted by switching the column selection valve for tandem flow over both immunodepletion columns, continuing flow of 100% Buffer B at 2 mL/min. Upon elution of the supermix bound fraction, both columns were neutralized and re-equilibrated by flowing 100% Buffer C (1X Seppro Neutralization Buffer, Sigma) in tandem over both columns at 2 mL/min for 18 min, followed by re-equilibration with 100% Buffer A for 20 min.

Digestion: pooled flow-through fractions (depleted fractions) from each sample were concentrated via Amicon filtration units (4 kDa MWCO), diluted with 10 mL 8 M urea in 100 mM EPPS (4-(2-Hydroxyethyl)-1-piperazinepropanesulfonic acid) pH 8.1, and re-concentrated in the same filtration unit to 300 μL. Concentrated samples were reduced with DTT (5 mM) for 1 h at room temperature, alkylated with Iodoacetamide (15 mM) for 1 h at room temperature in the dark, and quenched with DTT to 10 mM. Reduced and alkylated samples were digested with Lysyl endopeptidase (LysC, Wako Chemicals USA) at 1:25 enzyme:protein overnight at room temperature, diluted to 1 M urea with 100 mM EPPS pH 8.1, and digested with trypsin (Promega) at a ratio of 1:15 enzyme:protein for 6 h at 37 °C. Digestion efficiency of >90% was verified by micropurification (C18 STAGE tips) and fluorescence peptide assay (Thermo Scientific, Cat# 23290), followed by LC-MS analysis (35 min gradient) of 5% of 3 samples from each group of 10 samples.

TMT Labeling: digested samples were purified by C18 Sep Pak, and eluted samples were dried down via speedvac. Samples were resuspended in 100 μL of 100 mM EPPS pH 8.1 and labeled with 500 μg of TMT 11-Plex reagent (ThermoFisher). Labeling efficiency (>95% labeling efficiency) was verified by mixing 2% of each sample from each 10-plex, micropurification (C18 STAGE tips) and LC-MS analysis (85 min gradient). Based on the total intensity observed from each TMT channel in the labeling efficiency check, the remainder of each sample for each 10-plex was mixed such that total signal in each channel was approximately equal. The mixed sample was purified by C18 Sep Pak to remove the unreacted TMT, hydroxylamine, salts, and eluted peptides were concentrated via speedvac.

TMT-bridge normalization: the bridge sample was made by combining an equal aliquot of each sample and including that mixed sample within each plex. The bridge normalization is a ratio of each sample to the bridge sample. After normalizing for mixing errors (making the sum signal of all samples the same) each sample within each plex was normalized to the bridge channel by dividing by the bridge signal, such that <1 = lower than bridge and >1 = higher than the bridge.

Fractionation: labeled, mixed samples were fractionated by high pH reversed phase chromatography into 96 fractions, using an Agilent 1200 series HPLC system, and a 3 × 100 mM column packed with 1.9 µm C18 Poroshell material (Agilent). Chromatography was achieved by running a gradient of 5% Buffer B (10 mM ammonium bicarbonate, 90% acetonitrile) to 35% Buffer B in Buffer A (10 mM ammonium bicarbonate, 5% acetonitrile), over 60 min with timed fraction collection. These 96 fractions were pooled into 24 fractions. Fractions were dried via speedvac, cleaned by C18 micropurification, and analyzed via LC-MS.

LC-MS Analysis: peptide fractions were separated using a 120 min linear gradient from 8 to 25% acetonitrile in 0.1% formic acid. The MS was operated in a data dependent mode. The scan sequence began with FTMS1 spectra (resolution = 120,000; mass range of 350–1400 *m/z*; max injection time of 50 ms; Automatic Gain Control (AGC) target of 1e6; dynamic exclusion for 60 s with a +/−10 ppm window). The ten most intense precursor ions were selected for ITMS2 analysis via collisional-induced dissociation in the ion trap (normalized collision energy) = 35; max injection time = 100 ms; isolation window of 0.7 Da; AGC target of 2e4). Following ITMS2 acquisition, a synchronous-precursor-selection MS3 method was enabled to select 10 MS2 product ions for higher energy collisional-induced dissociation with analysis in the Orbitrap (normalized collision energy = 55; resolution = 50,000; max injection time = 110 ms; AGC target of 1.5e5; isolation window at 1.2 Da for +2 *m/z*, 1.0 Da for +3 *m/z* or 0.8 Da for +4 to +6 *m/z*).

All mass spectra were converted to mzXML using a modified version of ReAdW.exe. MS/MS spectra were searched against a concatenated 2018 human Uniprot protein database containing common contaminants (forward + reverse sequences) using the SEQUEST algorithm^49^. Database search criteria were as follows: fully tryptic with two missed cleavages; a precursor mass tolerance of 50 ppm and a fragment ion tolerance of 1 Da; oxidation of methionine (15.9949 Da) was set as differential modifications. Static modifications were iodoacetamide on cysteines (57.02146374) and TMT on lysines and N-termini of peptides (229.1629). Peptide-spectrum matches were filtered using linear discriminant analysis^50^ and adjusted to a 1% peptide false discovery rate (FDR)^51^ and collapsed further to a final 1.0% protein-level FDR. Peptides were assigned to proteins based on principles of parsimony. Peptides were assigned to the minimum set of proteins (IPI database entries) that could account for all peptides in the dataset. Proteins were quantified by summing the total reporter intensities across all matching PSMs.

IQP address: IQ Proteomics (http://www.iqproteomics.com/), LLC, 840 Memorial Drive, Cambridge, MA 02139.

### Targeted MS

The performance of candidate biomarkers in confirmatory and replication cohorts were monitored using internal standard triggered parallel reaction monitoring (SureQuant)-based relative quantification assays^52^. A minimum of three peptides per protein were selected for targeted assay development and the selection criteria were based on: 1. peptide abundance during discovery work, 2. protein unique sequence, 3. absence of methionine and cysteine residues, 4. no known modifications on amino acid residues, 5. no trypsin missed cleavages. The synthetic isotopically labeled lysine and arginine-^15^N, ^13^C ( ≥ 99% isotopic purity and ≥90% peptide purity) peptides were purchased from New England Peptides/Vivitide (https://vivitide.com/). Heavy labeled peptides were used as internal standard during the sample preparation and analysis. The list of selected peptides is reported in Supplementary Table 7.

### Quantitative proteomics workflow

Sample preparation: serum samples were depleted with a high select Top14 depletion resin (Fisher Scientific, Cat# A36372). A 600 μL of 50% of well mixed resin slurry was added into the each 96-well plate (Agilent PN 200957-100) followed by the addition of 30 μL serum samples and incubated for 15 min with gentle shaking. The use of Top14 resin allowed us to deplete the top 14 highly abundant proteins in human serum and improve the throughput of the process. The depleted samples were collected into the 96-well collection plate by centrifuging in a swinging bucket rotor for 2 min at 100 g. The protein eluant were then concentrated via Amicon filtration units (3 kDa MWCO) (Fisher Scientific, Cat# UFC500324). The protein concertation was measured using Micro BCA kit (Fisher Scientific, Cat# PI23235) and then adjusted to 1 μg/μL using 8 M urea in 100 mM EPPS pH 8.5. A 100 μg aliquot of protein from each sample were transferred into eppendorf tube followed by the addition of 400 mM DTT (Sigma Aldrich, Cat# D9779) to 5 mM. The disulfide bonds were reduced by incubating the samples for 1 h at room temperature shaking at 1000 rpm and were alkylated by the addition of 550 mM iodoacetamide with a final concentration of 15 mM (Sigma Aldrich, Cat# I1149) by incubating at 1 h in the dark. The alkylated protein samples were diluted to 1 M urea by the addition of 100 mM EPPS pH 8.5. Proteins were digested by adding lysyl endopeptidase (LysC, Wako Chemicals USA) at a ratio of 1:25 enzyme:protein overnight at room temperature. The second digestion was carried out using trypsin (Promega, Cat# V5111) at a ratio of 1:15 enzyme:protein for 6 h at 37 °C. After digestion, trypsin was inactivated by the addition of 10% formic acid to a final concentration of 0.5%. The tryptic peptides were concentrated and desalted with Strata™-X 33 µm Polymeric Reversed Phase 10 mg / well, 96-Well Plates (Phenomenex Cat# 8E-S100-AGB) according to the manufacturer’s instructions and were dehydrated to dryness in a speedvac.

### LC-MS/MS analysis

Digested peptides (1 μg) were analyzed by LC-MS/MS using a Dionex Ultimate 3000 UPLC (ThermoFisher Scientific) coupled online to an EASYSpray ion source and Exploris 480 MS (ThermoFisher Scientific). Peptides were resolved using an EASYSpray C18 reverse phase column (75 µm × 50 cm, ES803 Thermo Fisher Scientific) heated to 50 °C at a flow rate of 250 nL/min. A linear gradient of solvents A (0.1% formic acid in water) and B (0.1% formic acid, 90% acetonitrile) was run from 1–30% B over 30 min. Peptides were ionized at 1.7 kV and acquired on the Exploris 480 using SureQuant (ThermoFisher Scientific) internal standard triggered parallel reaction monitoring methodology^52^.

During the SureQuant runs, an MS1 scan from 400 *m/z* to 1000 *m/z* was acquired at 120,000 resolution with an AGC target of 3e6 and a maximum injection time of 50 ms. Following detection of a stable isotope labeled (SIL) peptide precursor above a minimum threshold (Supplementary Table 7 and 8) in the MS1 scan, a data-dependent MS2 scan of the SIL was acquired at a resolution of 15,000 with an AGC setting of 1e6, a maximum injection time of 20 ms, and a higher energy collisional-induced dissociation setting of 30%. Detection of 4 out of 6 prominent product ions (Supplementary Table 8) from the SIL resulted in a triggered acquisition of the light peptides via a data-dependent MS2 scan with an isolation offset dependent on the terminal amino acid and charge state of the SIL peptide at a resolution of 60,000 with an AGC setting of 1e6, a maximum injection time of 116 ms, and an higher energy collisional-induced dissociation setting of 30%. All spectra were acquired as profile. Design and analysis of the SureQuant targeted runs was performed in Skyline^53^. Manual inspection of all spectra was performed, and endogenous light peptide transitions were compared against the heavy standard in the same run. For endogenous peptides that were not detected in a sample, noise peaks in the retention time region of the heavy peptide were integrated to avoid missing values. Quantitation was reported as the light/heavy ratio of the most abundant transition for each peptide.

### Biomarker assays

Liver function tests for patients followed the local policy for current standard of clinical care at the respective recruiting centers via accredited medical laboratories. GLDH was evaluated using a Siemens Advia 1800 chemistry analyzer. CK18 and PCK2 were measured by SpectraMax 500 from Molecular Devices using CK18 M65 EpiDeath (Cat# P10040) and BioMatik (Cat# EKN47708) ELISA kits.

### Biomarker selection

For differential expression analysis of pairwise comparisons significant changes were defined by adjusted *p*-value: we did not set a threshold for FC. The identities of liver enriched genes were obtained from the human protein atlas (https://www.proteinatlas.org/)^13^.

Proteins identified during discovery phase were ranked by comparison statistics (cut-off: Benjamini-Hochberg adj *P* < 0.1), followed by the physiological function, interacting pathways, and tissue localization relevant to liver pathobiology. For short-listing, candidates were prioritized if reported as having either a causal association or a consequential relevance to liver disease following review against the current literature. To permit a ‘positive control approach’, traditional biomarkers ALT, AST and ALP, as well as previously identified biomarkers CK18 and GLDH^12,54^ were included.

### Statistical analysis

Discovery data analysis: TMT-bridge-normalized discovery data were transformed to log-scale using the “normalizeVSN” function of limma^55^. Differential expression analysis was then performed with limma using different pairwise comparisons (robust fit), after controlling for subject as a random effect using the “duplicateCorrelation” function and compensating for TMT channels using the “voomaByGroup” function. We considered Benjamini-Hochberg -adjusted *p*-values below 0.1 to be significant.

AUC-ROC and their confidence interval were calculated for each biomarker to evaluate the classification power and global predictivity of each biomarker in confirmatory and replication cohorts between DO versus HV, DO versus NDO, NDO versus HV, and DF patients who subsequently recovered (normalization of liver enzymes) (True recovery) versus those who did not (Not recovery) respectively (Supplementary Table 6). An AUC of 1.0 represents perfectly separable cases and controls (e.g. DO versus HV), while an AUC of 0.5 represents predictability no better than random guessing. Variable importance scores for candidate biomarkers were based on 500 bootstrapping. All analyses were conducted using R version 4.0.2.

To generate liver zone gene sets, we first obtained all significantly differentially expressed genes in hepatic cell cluster^14^, filtered for features with an average log2 fold difference >0, and then renamed clusters with liver zones as described by authors. Gene Set Enrichment Analysis was then performed using the fgsea package in R, with gene ranks from discovery data comparisons. We considered Benjamini-Hochberg-adjusted *p*-values below 0.1 to be significant.

### Reporting summary

Further information on research design is available in the Nature Portfolio Reporting Summary linked to this article.

## Supplementary information

Supplementary Information

Reporting Summary

## Data Availability

The discovery proteomics data generated in this study have been deposited in the MassIVE under massive.ucsd.edu with project identifier MSV000089782 [10.25345/C5R785S9H]. Uniprot protein database (https://www.uniprot.org) was used for protein identification from the mass spectrometry data. Targeted proteomics data is available through the Panorama repository via https://panoramaweb.org/DILI_Biomarkers.url and at the ProteomeXchange Consortium with following identifier PXD034882. All the clinical data used in the study are subject to a data sharing agreement with the originating institutions, available upon request to the corresponding authors, who will respond within 50 working days to provide contact details for contracting. Clinical data are available for research purposes only, for specified projects with reasonable security practices and systems in place to ensure confidentiality and data may not be shared with third parties. STARD checklist is provided in Supplementary Table 9. Source data are provided with this paper.

## Source data

Source Data

## Code availability

Code used to analyze the data in this study is available for use with an individual organization with adequate security measures to ensure confidentiality following provision of appropriate code sharing agreement with Pfizer. This can be arranged upon request to the corresponding authors, who will respond within 50 working days.
