# Supplementary material for: Tandem mass tag-based quantitative proteomic profiling identifies candidate serum biomarkers of drug-induced liver injury in humans
Source: Nat Commun. 2023 Mar 3;14:1215. doi: 10.1038/s41467-023-36858-6 (PMC9984368; doi:10.1038/s41467-023-36858-6)
Supplement: Supplementary file 1 — Supplementary Information [file 41467_2023_36858_MOESM1_ESM.pdf]

Supplementary Information

Tandem Mass Tag-Based Quantitative Proteomic Profiling Identifies Candidate Serum Biomarkers for the Diagnosis of Drug-Induced Liver Injury in Humans

Kodihalli C. Ravindra[1#], Vishal S. Vaidya[1#*], Zhenyu Wang[1], Joel D. Federspiel[1], Richard Virgen-Slane[1], Robert A. Everley[1], Jane I. Grove[2,3], Camilla Stephens[4,5], Mireia F. Ocana[1], Mercedes Robles-Díaz[4,5], M Isabel Lucena[4,5], Raul J. Andrade[4,5], Edmond Atallah[2,3], Alexander L. Gerbes[6], Sabine Weber[6], Helena Cortez-Pinto[7], Andrew J. Fowell[8], Hyder Hussaini[9], Einar S. Bjornsson[10,11], Janisha Patel[12], Guido Stirnimann[13], Sumita Verma[14], Ahmed M. Elsharkawy[15], William J.H. Griffiths[16], Craig Hyde[1], James W. Dear[17], Guruprasad P. Aithal[2,3,ϕ*] and Shashi K. Ramaiah[1,ϕ*]

# These authors contributed equally

ϕ These authors jointly supervised this work

* Corresponding authors: Vishal.Vaidya@Pfizer.com, Guru.Aithal@Nottingham.ac.uk, Shashi.Ramaiah@Pfizer.com

Running title: Candidate biomarkers for diagnosis of drug-induced liver injury

TABLE OF CONTENTS

SUPPLEMENTARY MATERIALS

1. Supplementary Figures

- Supplementary Figure 1: Comparison of leukocyte cell-derived chemotaxin 2 (LECT2) levels in the discovery cohort.
- Supplementary Figure 2: Differential expression of candidate biomarkers between DO, DF, NDO, and NDF in the confirmatory cohort.
- Supplementary Figure 3: Differential expression of mitochondrial phosphoenolpyruvate carboxykinase 2 (PCK2).
- Supplementary Figure 4: Development of models to distinguish DO from HV
- Supplementary Figure 5: Development of models to distinguish DO from NDO
- Supplementary Figure 6: Gene signature analysis using the liver cell population for differentially expressed liver enriched proteins.
- Supplementary Figure 7: Correlation between ALT activity and candidate biomarkers (HPD, OTC, GSTA1, DMGDH, CES1, LECT2, and PCK2).

2. Supplementary Tables

- Supplementary Table 1: Demographics and clinical characteristics of the three study cohorts.
- Supplementary Table 2: Area under the receiver operator characteristic curve (AUC) for candidate biomarkers for DO versus HV, and NDO versus DO in the confirmatory cohort.
- Supplementary Table 3: Area under the receiver operator characteristic curve (AUC) for candidate biomarkers for DO versus HV, and NDO versus DO in the replication cohort.
- Supplementary Table 4: Summary results from candidate, previously identified, and traditional biomarker multivariate models at a fixed specificity and sensitivity for the diagnosis of liver injury (NDO versus DO).
- Supplementary Table 5: Assessment of the Logistic Regression and Random Forest candidate, previously identified, and traditional biomarker models in the confirmatory and replication cohorts.
- Supplementary Table 6: Area under the receiver operator characteristic curve (AUC-ROC) for candidate biomarkers between complete recovery versus partial recovery in confirmatory and replication cohort.
- Supplementary Table 7: Peptides list for all target proteins used for targeted MS assay.
- Supplementary Table 8: Stable isotope labelled (SIL) peptide precursor and minimum threshold in the MS1 scan.
- Supplementary Table 9: STARD checklist.

Supplementary Figures

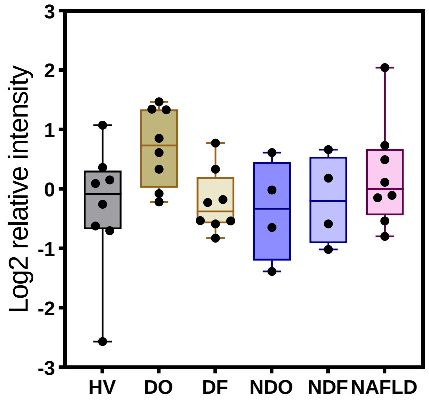

Supplementary Figure 1. **Comparison of leukocyte cell-derived chemotaxin 2 (LECT2) levels in the discovery cohort.** The box and whisker plots represent the levels of LECT2 in the discovery cohort comprising healthy volunteers (HV, *n* = 10), acute DILI onset (DO, *n* = 10), DILI follow-up (DF, *n* = 10), acute non-DILI onset (NDO, *n* = 5), non-DILI follow-up (NDF *n* = 5), and chronic non-alcoholic fatty liver disease (NAFLD, *n* = 10). The centre line in the box corresponds to the median, the box represents the first and third quartiles, and the whiskers represent the minimum and maximum observed values. Source data are provided as a Source Data file.

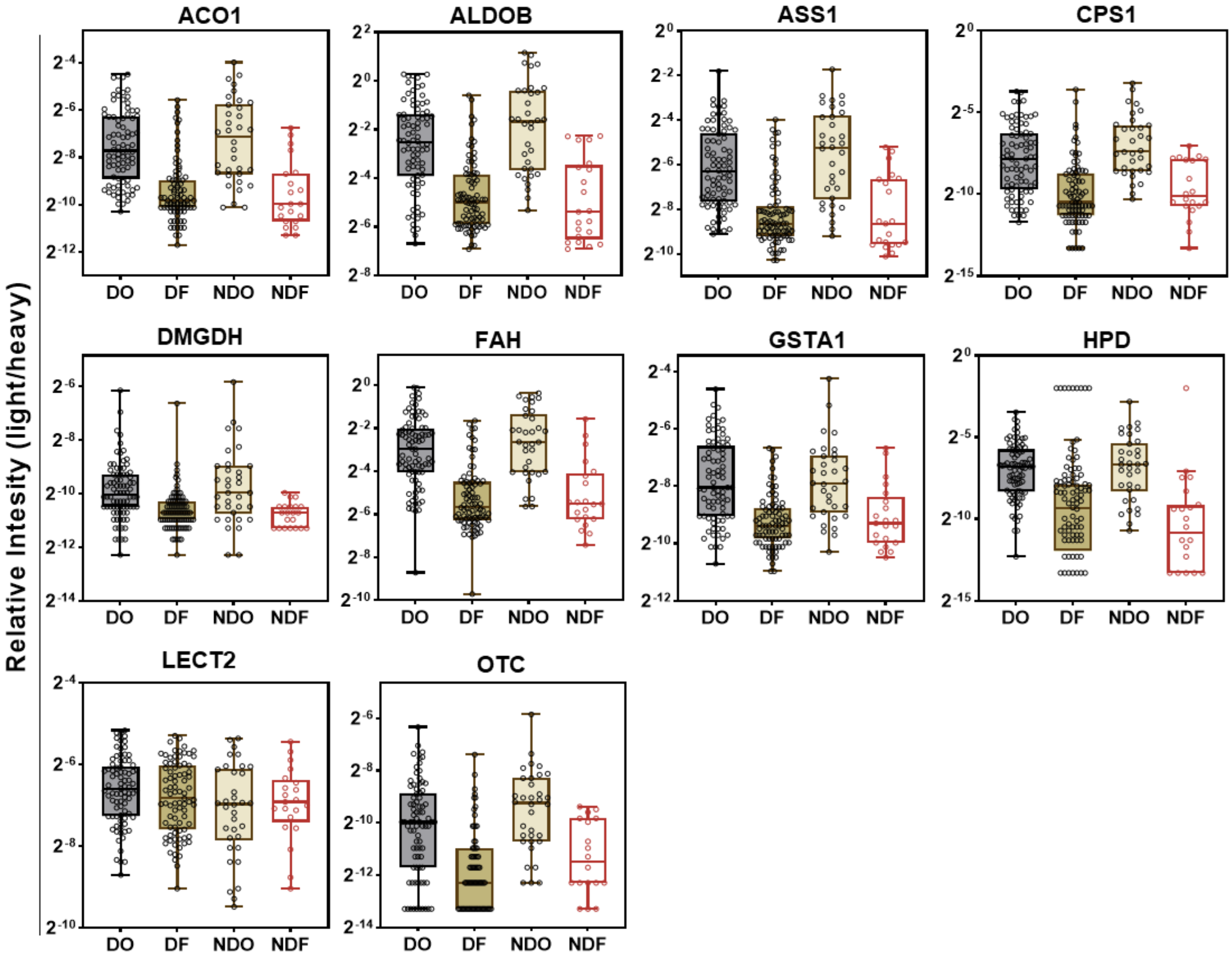

Supplementary Figure 2. Differential expression of candidate biomarkers between DO, DF, NDO, and NDF in the confirmatory cohort. Relative quantities of candidate biomarkers: cytoplasmic aconitate hydratase (ACO1), fructosebisphosphate aldolase B (ALDOB), argininosuccinate synthase (ASS1), carbamoylphosphate synthase (CPS1), mitochondrial dimethylglycine dehydrogenase (DMGDH), fumarylacetoacetase (FAH), glutathione S-transferase A1 (GSTA1), 4-hydroxyphenylpyruvate dioxygenase (HPD), leukocyte cell-derived chemotaxin 2 (LECT2), ornithine carbamoyltransferase (OTC) in the confirmatory cohort individuals (DO (n = 82), DF (n = 77), NDO (n = 34) and NDF (n = 22)). The centre line in the box corresponds to the median, the box represents the first and third quartiles, and the whiskers represent the minimum and maximum observed values. Source data are provided as a Source Data file.

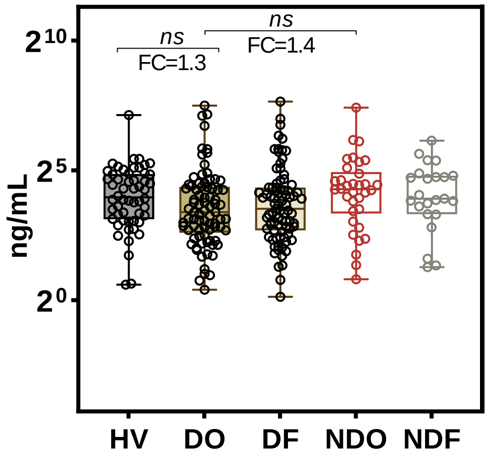

Supplementary Figure 3. Differential expression of mitochondrial phosphoenolpyruvate carboxykinase 2, (PCK2). The absolute concentration of PCK2 in individual HV (n = 60), DO (n = 82), DF (n = 77), NDO (n = 34) and NDF (n = 22) within the confirmatory cohort. The centre line in the box corresponds to the median, the box represents the first and third quartiles, and the whiskers represent the minimum and maximum observed values. Fold change (FC) and statistical test (t test, two-sided, no adjustment) outcome are shown (ns = not significant). Source data are provided as a Source Data file.

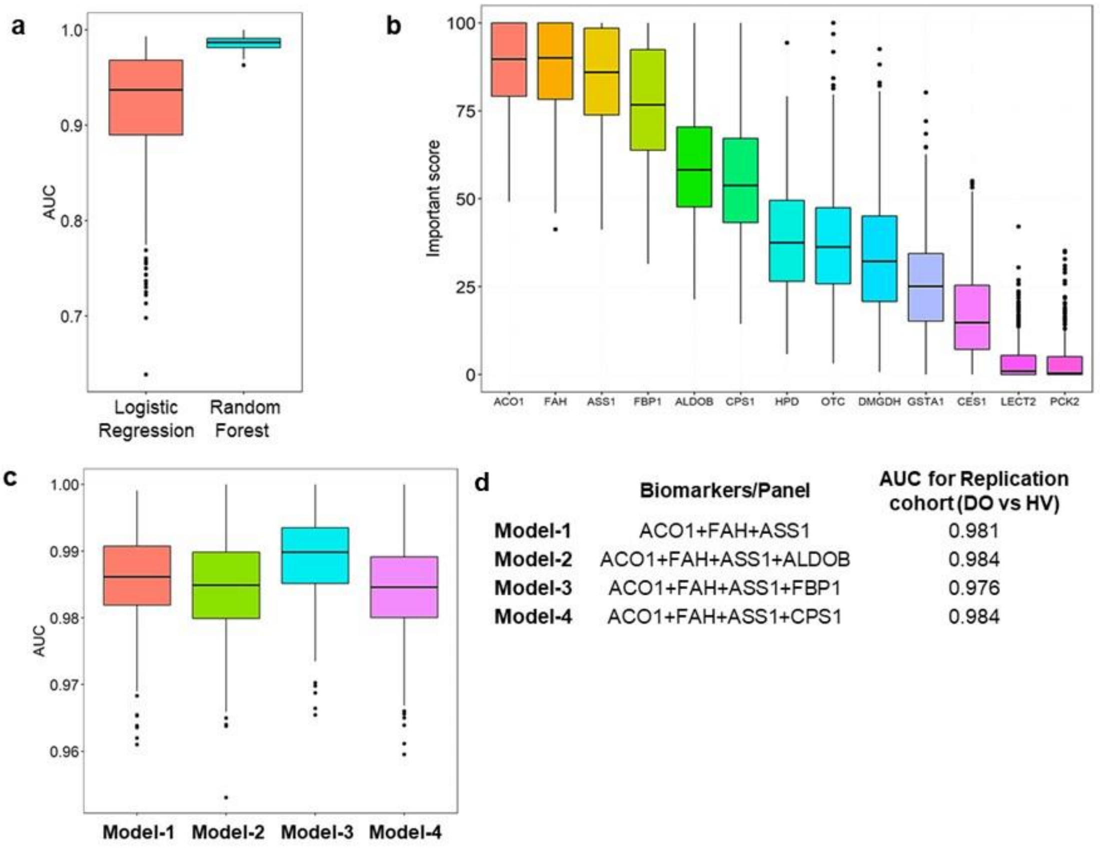

	Biomarkers/Panel	AUC for Replication cohort (DO vs HV)
**Model-1**	ACO1+FAH+ASS1	0.981
**Model-2**	ACO1+FAH+ASS1+ALDOB	0.984
**Model-3**	ACO1+FAH+ASS1+FBP1	0.976
**Model-4**	ACO1+FAH+ASS1+CPS1	0.984

Supplementary Figure 4. Development of multivariate models to distinguish DO from HV. a AUC from logistic regression and random forest (RF) predictive, multivariate models including all candidate biomarkers using confirmatory cohort, DO (*n* = 76) and HV (*n* = 60). Box plots indicate median (middle line), 25th, 75th percentile (box) and 95th percentile (whiskers) as well as outliers (single points). **b** Variable importance scores for candidate biomarkers based on 500 bootstrapping in RF model from (**a**); The y-axis represents the importance scores scaled to a maximum score of 100. Box plots indicate median (middle line), distribution of score (box) and 1.5x interquartile range (whiskers) as well as outliers (single points) which may be truncated by axes limits at 0 or 100. **c** AUC of 4 RF models or panels and each model is described in (**d**). Box plots indicate median (middle line), 25th, 75th percentile (box) and 95th percentile (whiskers) as well as outliers (single points). **d** AUCs for the 4 models in (**c**) tested using the replication cohort as an independent validation dataset. Source data are provided as a Source Data file.

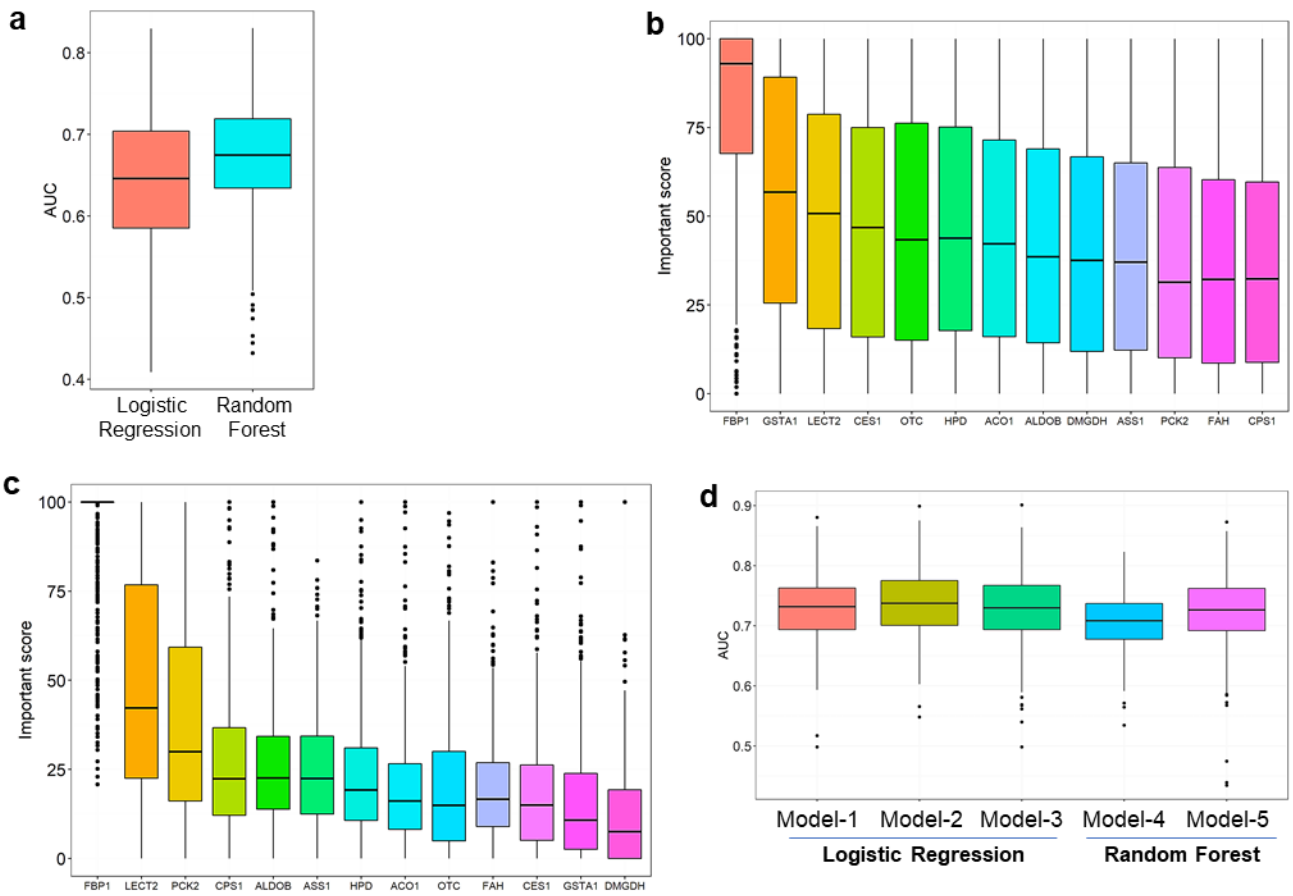

Supplementary Figure 5. Development of models to distinguish DO from NDO. a AUC from logistic regression and random forest (RF) predictive, multivariate models including all candidate biomarkers using confirmatory cohort, NDO (*n* = 32) and DO (n = 76). **b** and **c** Variable importance scores for candidate biomarkers based on 500 bootstrapping, logistic regression **(b)** and RF **(c)** from NDO and DO in (**a**). The y-axis represents the importance scores scaled to a maximum score of 100. **d** AUC for logistic regression and RF models (shown in Table 2) developed based on the best performing biomarkers (Supplemental Tables 4 and 5) Model 1: FBP1+GSTA1; Model 2: FBP1+GSTA1+LECT2; Model 3: FBP1+CES1+LECT2; Model 4: FBP1+LECT2; Model 5: FBP1+LECT2+CPS1. Box plots indicate median (middle line), distribution of score (box) and 1.5x interquartile range (whiskers) as well as outliers (single points) which may be truncated by axes limits at 0 or 100. In **(a)** and (**d**), box plots indicate median (middle line), 25th, 75th percentile (box) and 95th percentile (whiskers) as well as outliers (single points). Source data are provided as a Source Data file.

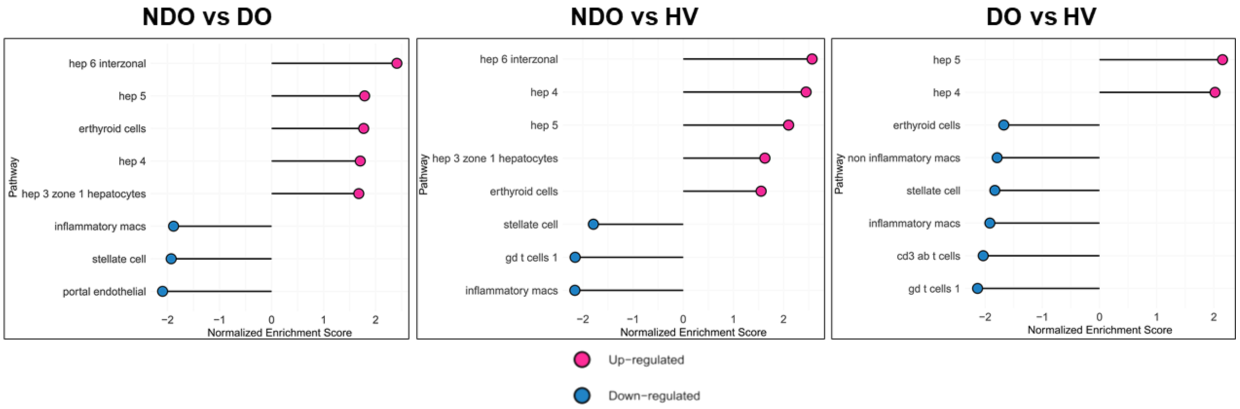

Supplementary Figure 6. Gene signature analysis using the liver cell population for differentially expressed liver enriched proteins. The pathway enrichment scores for pairwise comparison between NDO and DO, NDO and HV, DO and HV are shown to identify up- or down-regulated pathways in liver zones. The X-axis represents normalized enrichment scores, calculated by the fgsea package for the pathways shown on the Y-axis.

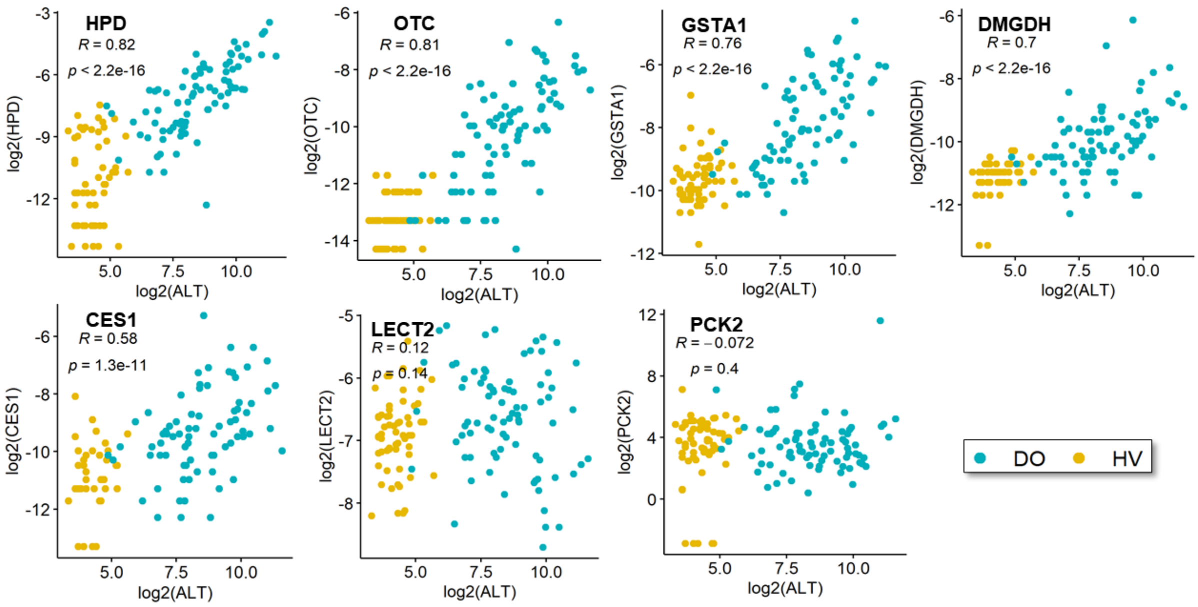

Supplementary Figure 7. Correlation between ALT activity and candidate biomarkers (HPD, OTC, GSTA1, DMGDH, CES1, LECT2, and PCK2). The individual log2 normalized levels, correlation coefficient and significance levels (two-sided, no adjustment) are shown for confirmatory cohort HV (n = 60) and patients with onset of DILI (DO, n = 82). Source data are provided as a Source Data file.

Supplementary Tables

Supplementary Table 1. Demographics and clinical characteristics of the three study cohorts.

	**Discovery cohort**				**Confirmatory cohort**			**Replication cohort**		
	**HV** *n*=10	**DILI** *n*=10	**Non-DILI** *n*=5	**NAFLD** *n*=10	**HV** *n*=60	**DILI** *n*=82	**Non-DILI** *n*=34	**HV** *n*=34	**DILI** *n*=41	**Non-DILI** *n*=24
**Age (years), mean ±SD**	57 ±13	60 ±17	61 ±15	58 ±15	51 ±13	54 ±18	51 ±20	41 ±17	56 ±17	53 ±17
**Sex, male/female (%)**	60/40	60/40	20/80	60/40	32/68	56/46	38/62	36/64	27/73	42/58
**BMI (Kg/m^2), mean ±SD**	26.3 ±5.3	26.6 ±4.2	26.4 ±6.0	34.3 ±5.1	ND	25.8 ±4.8	25.5 ±4.3	25.3 ±5.1	25.4 ±5.5	27.9 ±5.5
**AHT, (%)**	ND	33	40	50	ND	30	24	ND	24	25
**DM, (%)**	ND	10	0	60	ND	12	8.8	ND	7.3	4.2
**Dyslipidemia, (%)**	ND	30	40	ND	ND	20	12	ND	32	12
**Jaundice, (%)**	-	60	60	-	-	52	85	-	66	62
**Hospitalization, (%)**	-	30	40	-	-	67	76	-	80	71
**Pattern of liver injury, (%)**										
**Hepatocellular**	-	22	40	-	-	63	74	-	52	67
**Cholestatic**	-	44	40	-	-	25	18	-	28	29
**Mixed**	-	33	20	-	-	12	8.8	-	20	4.2
**Severity, (%)**										
**Mild**	-	40	40	-	-	36	8.8	-	27	29
**Moderate**	-	60	60	-	-	57	56	-	61	58
**Severe**	-	0	0	-	-	4.9	24	-	7.3	8.3
**Fatal/ LT**	-	0	0	-	-	2.5	12	-	4.9	4.2

AHT, arterial hypertension; BMI, body mass index, DM, diabetes mellitus (type 1 or 2); LT, liver transplantation; ND, no data available; SD, standard deviation.

Supplementary Table 2. Area under the receiver operator characteristic curve (AUC) for candidate biomarkers for DO versus HV, and NDO versus DO in the confirmatory cohort.

Biomarker	DO vs HV		NDO vs DO	
	AUC	95% CI	AUC	95% CI
ALT	1.00	0.99 – 1	0.63	0.51 – 0.75
AST	0.99	0.98 – 1	0.64	0.52 – 0.75
ALP	0.93	0.89 – 0.97	0.53	0.42 – 0.63
TBL	0.92	0.87 – 0.96	0.65	0.55 – 0.76
GLDH	0.86	0.79 – 0.92	0.48	0.36 – 0.59
CK18	0.96	0.92 – 0.99	0.66	0.54 – 0.77
ACO1	0.99	0.98 – 1	0.54	0.42 – 0.66
ASS1	0.98	0.97 – 1	0.59	0.47 – 0.71
FAH	0.98	0.95 – 1	0.56	0.44 – 0.68
FBP1	0.96	0.93 – 1	0.75	0.64 – 0.86
CPS1	0.96	0.93 – 0.99	0.61	0.50 – 0.72
ALDOB	0.94	0.91 – 0.98	0.60	0.48 – 0.72
HPD	0.94	0.90 – 0.97	0.53	0.41 – 0.65
OTC	0.92	0.88 – 0.96	0.61	0.49 – 0.72
GSTA1	0.87	0.81 – 0.93	0.48	0.37 – 0.60
DMGDH	0.86	0.80 – 0.92	0.52	0.40 – 0.65
CES1	0.80	0.71 – 0.88	0.47	0.34 – 0.59
LECT2	0.61	0.52 – 0.70	0.62	0.50 – 0.74
PCK2	0.56	0.46 – 0.66	0.63	0.52 – 0.75

ALT, AST, ALP, and TBL markers were used for defining acute DILI or non-DILI as described in methods section of the manuscript. GLDH and CK18 have previously been investigated and identified as promising biomarkers, so were included in our study.CI, confidence interval.

Supplementary Table 3. Area under the receiver operator characteristic curve (AUC) for candidate biomarkers for DO versus HV, and NDO versus DO in the replication cohort.

Biomarker	DO vs HV		NDO vs DO	
	AUC	95% CI	AUC	95% CI
ALT	1.00	0.99 – 1	0.57	0.42 – 0.72
AST	0.97	0.93 – 1	0.65	0.50 – 0.79
ALP	0.95	0.91 – 1	0.58	0.43 – 0.73
TBL	0.87	0.78 – 0.96	0.56	0.41 – 0.71
GLDH	0.84	0.74 – 0.94	0.48	0.32 – 0.64
CK18	0.97	0.94 – 1	0.65	0.51 – 0.78
ACO1	0.98	0.95 – 1	0.61	0.47 – 0.75
ASS1	0.97	0.93 – 1	0.57	0.43 – 0.72
FAH	0.99	0.97 – 1	0.64	0.50 – 0.78
FBP1	0.94	0.88 – 0.99	0.65	0.52 – 0.79
CPS1	0.95	0.91 – 1	0.64	0.50 – 0.78
ALDOB	0.95	0.90 – 1	0.60	0.46 – 0.74
HPD	0.96	0.92 – 1	0.60	0.45 – 0.75
OTC	0.92	0.87 – 0.98	0.58	0.44 – 0.72
GSTA1	0.93	0.87 – 0.98	0.56	0.42 – 0.71
DMGDH	0.73	0.61 – 0.84	0.55	0.41 – 0.70
CES1	0.80	0.70 – 0.90	0.63	0.49 – 0.77
LECT2	0.58	0.45 – 0.72	0.54	0.40 – 0.69

Supplementary Table 4. Summary results from candidate, previously identified, and traditional biomarker multivariate models at a fixed specificity and sensitivity for the diagnosis of liver injury (NDO versus DO).

Matric	Model	Threshold	Confirmatory cohort						Replication cohort					
**Specificity > 0.90**			Specificity	Sensitivity	TN	TP	FN	FP	Specificity	Sensitivity	TN	TP	FN	FP
Logistic Regression	FBP1+GSTA1+AST+TBL+ALT	0.49	0.92	0.47	66	14	16	6	0.82	0.26	31	6	17	7
	FBP1+GSTA1+LECT2+AST+ALT+TBL	0.45	0.90	0.57	65	17	13	7	0.79	0.35	30	8	15	8
	FBP1+CES1+LECT2+ALT+AST+TBL	0.54	0.93	0.53	67	16	14	5	0.79	0.22	30	5	18	8
Random Forest	FBP1+LECT2+TBL+AST	0.45	1.00	1.00	72	30	0	0	0.74	0.39	28	9	14	10
	FBP1+LECT2+CPS1+TBL+AST+CK18+ALT	0.46	1.00	1.00	72	30	0	0	0.74	0.43	28	10	13	10
**Sensitivity > 0.90**														
Logistic Regression	FBP1+GSTA1+AST+TBL+ALT	0.14	0.46	0.93	33	28	2	39	0.39	0.83	15	19	4	23
	FBP1+GSTA1+LECT2+AST+ALT+TBL	0.15	0.50	0.90	36	27	3	36	0.50	0.74	19	17	6	19
	FBP1+CES1+LECT2+ALT+AST+TBL	0.15	0.49	0.90	35	27	3	37	0.47	0.78	18	18	5	20
Random Forest	FBP1+LECT2+TBL+AST	0.45	1.00	1.00	72	30	0	0	0.74	0.39	28	9	14	10
	FBP1+LECT2+CPS1+TBL+AST+CK18+ALT	0.46	1.00	1.00	72	30	0	0	0.74	0.43	28	10	13	10

TN, true negative; TP, true positive; FN, false negative; FP, false positive.

Supplementary Table 5. Assessment of the logistic regression and random forest models in the confirmatory and replication cohorts.

Method	Biomarkers/Models	AUC of confirmatory cohort between NDO vs DO	AUC of replication cohort between NDO vs DO
Logistic Regression	FBP1+GSTA1+AST+TBL+ALT	0.79	0.67
	FBP1+GSTA1+LECT2+AST+ALT+TBL	0.80	0.67
	FBP1+CES1+LECT2+ALT+AST+TBL	0.80	0.66
Random Forest	FBP1+LECT2+TBL+AST	1.00	0.61
	FBP1+LECT2+CPS1+TBL+AST+CK18+ALT	1.00	0.60

Supplementary Table 6. Area under the receiver operator characteristic curve (AUC) for candidate biomarkers between complete recovery versus partial recovery in confirmatory and replication cohort.

Biomarker	Confirmatory		Replication	
	AUC	95% CI	AUC	95% CI
ALT	0.91	0.84 – 0.97	0.99	0.97 – 1
AST	0.88	0.81 – 0.96	0.97	0.89 – 1
ALP	0.91	0.84 – 0.97	0.84	0.70 – 0.98
TBL	0.73	0.61 – 0.85	0.89	0.76 – 1
GLDH	0.80	0.68 – 0.92	0.90	0.79 – 1
CK18	0.68	0.56 – 0.81	0.94	0.84 – 1
ACO1	0.79	0.68 – 0.91	0.93	0.83 – 1
ASS1	0.86	0.76 – 0.97	0.87	0.73 – 1
FAH	0.74	0.62 – 0.86	0.95	0.86 – 1
CPS1	0.85	0.74 – 0.96	0.85	0.70 – 1
ALDOB	0.82	0.70 – 0.94	0.86	0.71 – 1
HPD	0.69	0.54 – 0.85	0.79	0.63 – 0.95
OTC	0.74	0.59 – 0.88	0.92	0.82 – 1
GSTA1	0.63	0.49 – 0.79	0.83	0.67 – 1
DMGDH	0.70	0.59 – 0.82	0.75	0.58 – 0.92
LECT2	0.53	0.38 – 0.69	0.58	0.35 – 0.80
FBP1	nd		0.86	0.73 – 0.99
PCK2	0.57	0.42 – 0.72	nd	
CES1	nd		0.80	0.64 – 0.96

CI, confidence interval; nd, biomarker was not determined.

Supplementary Table 7. Peptides list for all target proteins used for targeted MS assay.

Gene name	Sequence	Number of AAs	MW
ALDOB	H2N-ELSEIAQSIVANG**K**^-OH	14	1466.6
	H2N-ALQASALAAWGG**K**^-OH	13	1251.4
	H2N-LDQGGAPLAGTN**K**^-OH	13	1249.4
ASS1	H2N-YVSHGATG**K**^-OH	9	927.0
	H2N-NQAPPGLYT**K**^-OH	10	1096.2
	H2N-GQVYILG**R**^-OH	8	915.1
CPS1	H2N-AQTAHIVLEDGT**K**^-OH	13	1390.5
	H2N-TFEESFQ**K**^-OH	8	1023.1
	H2N-GQNQPVLNITN**K**^-OH	12	1333.5
ACO1	H2N-AVLAESYE**R**^-OH	9	1047.1
	H2N-GFQVAPEHHNDH**K**^-OH	13	1523.6
	H2N-VLLEAAI**R**^-OH	8	894.1
DMDGH	H2N-DGLLFGPYESQE**K**^-OH	13	1490.6
	H2N-LEEETGQVVGFHQPGSI**R**^-OH	18	1993.2
	H2N-VAVTDLSPFG**K**^-OH	11	1141.3
FAH	H2N-HLFTGPVLS**K**^-OH	10	1106.3
	H2N-LGEPIPIS**K**^-OH	9	961.1
	H2N-ASSVVVSGTPI**R**^-OH	12	1182.3
GSTA1	H2N-LHYFNA**R**^-OH	7	930.0
	H2N-SHGQDYLVGN**K**^-OH	11	1225.3
	H2N-AILNYIAS**K**^-OH	9	1000.2
HPD	H2N-AFEEEQNL**R**^-OH	9	1145.2
	H2N-EVVSHVI**K**^-OH	8	918.1
	H2N-EPWVEQD**K**^-OH	8	1038.1
LECT2	H2N-NAINNGV**R**^-OH	8	866.9
	H2N-LGTLLPLQ**K**^-OH	9	990.2
OTC	H2N-GYEPDASVT**K**^-OH	10	1074.1
	H2N-NFTGEEI**K**^-OH	8	945.0
	H2N-SLVFPEAEN**R**^-OH	10	1171.3
FBP1	H2N-EAVLDVIPTDIHQ**R**^-OH	14	1616.8
	H2N-DALQPG**R**^-OH	7	765.8
	H2N-DFDPAVTEYIQ**R**^-OH	12	1463.6
CES1	H2N-EGYLQIGANTQAAQ**K**^-OH	15	1599.7
	H2N-ELIPEATE**K**^-OH	9	1037.2
	H2N-FLSLDLQGDP**R**^-OH	11	1270.4

K: $^{13}C_6$, $^{15}N_2$ labelled lysine; **R**: $^{13}C_6$, $^{15}N_4$ labelled arginine. AAs, amino acids; MW, molecular weight.

Supplementary Table 8. Stable isotope labelled (SIL) peptide precursor and minimum threshold in the MS1 scan.

Peptide Sequence	m/z	MS1 Intensity Threshold	Charge State	Isolation offset (m/z)
GQVYILGR	458.268	310000	2	-5.004
VLLEAAIR	447.786	1400000	2	-5.004
AVLAESYER	524.271	1000000	2	-5.004
ASSVVVSGTPIR	591.84	240000	2	-5.004
LHYFNAR	465.745	67000	2	-5.004
AFEEEQNLR	573.277	950000	2	-5.004
NAINNGVR	434.237	650000	2	-5.004
SLVFPEAENR	586.303	1400000	2	-5.004
AQIFANTVDNAR	665.343	260000	2	-5.004
LEAEIATYR	538.287	2400000	2	-5.004
DALQPGR	383.7077	50000	2	-5.004
DFDPAVTEYIQR	732.3557	100000	2	-5.004
FLSLDLQGDPR	635.8369	100000	2	-5.004
ELSEIAQSIVANGK	733.901	460000	2	-4.007
LDQGGAPLAGTNK	625.335	310000	2	-4.007
ALQASALAAWGGK	626.35	280000	2	-4.007
NQAPPGLYTK	548.798	360000	2	-4.007
GQNQPVLNITNK	667.369	410000	2	-4.007
TFEESFQK	512.247	1100000	2	-4.007
DGLLFGPYESQEK	745.866	2200000	2	-4.007
VAVTDLSPFGK	571.321	1700000	2	-4.007
HLFTGPVLSK	553.826	1100000	2	-4.007
LGEPIPISK	481.294	260000	2	-4.007
AILNYIASK	500.8	140000	2	-4.007
SHGQDYLVGNK	613.306	55000	2	-4.007
EVVSHVIK	459.779	98000	2	-4.007
EPWVEQDK	519.753	1700000	2	-4.007
LGTLLPLQK	495.826	800000	2	-4.007
NFTGEEIK	473.242	380000	2	-4.007
GYEPDASVTK	537.763	420000	2	-4.007
NYTDNELEK	567.264	750000	2	-4.007
DWSHYFK	495.732	50000	2	-4.007
EGYLQIGANTQAAQK	800.4145	100000	2	-4.007
ELIPEATEK	519.2839	50000	2	-4.007
LEEETGQVVGFHQPGSIR	665.005	1600000	3	-3.336
EAVLDVIPTDIHQR	539.2941	10000	3	-3.336
AQTAHIVLEDGTK	464.252	1100000	3	-2.671
GFQVAPEHHNDHK	508.579	180000	3	-2.671

Supplementary Table 9: STARD checklist

Section & Topic	No	Item	Reported on page #
**TITLE OR ABSTRACT**			
	**1**	Identification as a study of diagnostic accuracy using at least one measure of accuracy (such as sensitivity, specificity, predictive values, or AUC)	abstract
**ABSTRACT**			
	**2**	Structured summary of study design, methods, results, and conclusions (for specific guidance, see STARD for Abstracts)	abstract
**INTRODUCTION**			
	**3**	Scientific and clinical background, including the intended use and clinical role of the index test	Introduction
	**4**	Study objectives and hypotheses	Introduction
**METHODS**			
Study design	**5**	Whether data collection was planned before the index test and reference standard were performed (prospective study) or after (retrospective study)	Methods
Participants	**6**	Eligibility criteria	Methods
	**7**	On what basis potentially eligible participants were identified (such as symptoms, results from previous tests, inclusion in registry)	Methods
	**8**	Where and when potentially eligible participants were identified (setting, location and dates)	Methods
	**9**	Whether participants formed a consecutive, random or convenience series	Methods
Test methods	**10a**	Index test, in sufficient detail to allow replication	Methods
	**10b**	Reference standard, in sufficient detail to allow replication	Methods
	**11**	Rationale for choosing the reference standard (if alternatives exist)	Methods
	**12a**	Definition of and rationale for test positivity cut-offs or result categories of the index test, distinguishing pre-specified from exploratory	Results
	**12b**	Definition of and rationale for test positivity cut-offs or result categories of the reference standard, distinguishing pre-specified from exploratory	na
	**13a**	Whether clinical information and reference standard results were available to the performers/readers of the index test	Methods
	**13b**	Whether clinical information and index test results were available to the assessors of the reference standard	Results
Analysis	**14**	Methods for estimating or comparing measures of diagnostic accuracy	Results
	**15**	How indeterminate index test or reference standard results were handled	Methods
	**16**	How missing data on the index test and reference standard were handled	Methods
	**17**	Any analyses of variability in diagnostic accuracy, distinguishing pre-specified from exploratory	Discussion
	**18**	Intended sample size and how it was determined	na
**RESULTS**			
Participants	**19**	Flow of participants, using a diagram	Fig 1
	**20**	Baseline demographic and clinical characteristics of participants	Table 1
	**21a**	Distribution of severity of disease in those with the target condition	na
	**21b**	Distribution of alternative diagnoses in those without the target condition	Table 1
	**22**	Time interval and any clinical interventions between index test and reference standard	Table 1
Test results	**23**	Cross tabulation of the index test results (or their distribution) by the results of the reference standard	na
	**24**	Estimates of diagnostic accuracy and their precision (such as 95% confidence intervals)	na
	**25**	Any adverse events from performing the index test or the reference standard	na

**DISCUSSION**			
	**26**	Study limitations, including sources of potential bias, statistical uncertainty, and generalisability	18
	**27**	Implications for practice, including the intended use and clinical role of the index test	19
**OTHER INFORMATION**			
	**28**	Registration number and name of registry	na
	**29**	Where the full study protocol can be accessed	Methods
	**30**	Sources of funding and other support; role of funders	Acknwledgements
