# Supplementary material for: Tandem mass tag-based quantitative proteomic profiling identifies candidate serum biomarkers of drug-induced liver injury in humans
Source: Nat Commun. 2023 Mar 3;14:1215. doi: 10.1038/s41467-023-36858-6 (PMC9984368; doi:10.1038/s41467-023-36858-6)
Supplement: Supplementary file 2 — Reporting Summary [file 41467_2023_36858_MOESM2_ESM.pdf]

nature portfolio

Corresponding author(s): Vishal Vaidya, Guruprasad Aithal, Shashi Ramaiah

Last updated by author(s): 01/25/2023

Reporting Summary

Nature Portfolio wishes to improve the reproducibility of the work that we publish. This form provides structure for consistency and transparency in reporting. For further information on Nature Portfolio policies, see our Editorial Policies and the Editorial Policy Checklist.

Statistics

For all statistical analyses, confirm that the following items are present in the figure legend, table legend, main text, or Methods section.

n/a	Confirmed	
☐	☒	The exact sample size (*n*) for each experimental group/condition, given as a discrete number and unit of measurement
☒	☐	A statement on whether measurements were taken from distinct samples or whether the same sample was measured repeatedly
☐	☒	The statistical test(s) used AND whether they are one- or two-sided<br>*Only common tests should be described solely by name; describe more complex techniques in the Methods section.*
☒	☐	A description of all covariates tested
☒	☐	A description of any assumptions or corrections, such as tests of normality and adjustment for multiple comparisons
☐	☒	A full description of the statistical parameters including central tendency (e.g. means) or other basic estimates (e.g. regression coefficient) AND variation (e.g. standard deviation) or associated estimates of uncertainty (e.g. confidence intervals)
☐	☒	For null hypothesis testing, the test statistic (e.g. *F*, *t*, *r*) with confidence intervals, effect sizes, degrees of freedom and *P* value noted<br>*Give P values as exact values whenever suitable.*
☒	☐	For Bayesian analysis, information on the choice of priors and Markov chain Monte Carlo settings
☐	☒	For hierarchical and complex designs, identification of the appropriate level for tests and full reporting of outcomes
☐	☒	Estimates of effect sizes (e.g. Cohen's *d*, Pearson's *r*), indicating how they were calculated

Our web collection on statistics for biologists contains articles on many of the points above.

Software and code

Policy information about availability of computer code

Data collection	No specific software was used during trial data collection
Data analysis	SEQUEST algorithm (Eng JK, J Am Soc Mass Spectrom-1994), SKYLINE version 21.2 - 64 bit (MacLean B, Bioinformatics-2010). The software code used in this study is available upon request.

For manuscripts utilizing custom algorithms or software that are central to the research but not yet described in published literature, software must be made available to editors and reviewers. We strongly encourage code deposition in a community repository (e.g. GitHub). See the Nature Portfolio guidelines for submitting code & software for further information.

Data

Policy information about availability of data

All manuscripts must include a data availability statement. This statement should provide the following information, where applicable:

- Accession codes, unique identifiers, or web links for publicly available datasets
- A description of any restrictions on data availability
- For clinical datasets or third party data, please ensure that the statement adheres to our policy

The discovery proteomics data generated in this study have been deposited in the MassIVE under massive.ucsd.edu with project identifier MSV000089782 [ftp://massive.ucsd.edu/MSV000089782/]. Uniprot protein database (https://www.uniprot.org) was used for protein identification from the mass spectrometry data. Targeted proteomics data is available through the Panorama repository via https://panoramaweb.org/DILI_Biomarkers.url and at the ProteomeXchange Consortium with following identifier PXD034882. All the clinical data presented are available upon reasonable request to the corresponding authors with appropriate data

sharing agreements.

Human research participants

Policy information about [studies involving human research participants and Sex and Gender in Research.](#)

Reporting on sex and gender	Both sexes were included in the study design as this is a rare disease affecting both sexes. Sex data was collected based on self reporting. Proportions in sub-groups is reported but sex-based analyses could not be performed due to small size of cohort. We did not have consent for sharing individual data since this allows potential for individual identification based on phenotypic features within a small cohort despite recruitment at multiple centres across Europe.
Population characteristics	Table 1 in manuscript
Recruitment	The two key groups that we have compared, DILI and acute non-DILI controls, were consecutive patients that were referred to secondary or tertiary care health services in Europe. These are provided either through wither public services (UK) or insurance systems or combination in other countries in Europe. These may influence how early patients present, but, unlikely to affect the two comparative groups differently. Therefore, the results related to biomarkers are unlikely to be affected by self-selection or other biases<br>NAFLD controls were recruited consecutively through secondary care services in the Nottingham University Hospitals NHS Trust. All these patients are referred from primary care which is provided as a public service. We enrolled consecutive patients from a large secondary service population. It is possible that health conscious/ or individuals with health seeking behavior are more likely to seek routine health checks at primary care level and it is possible that there is an element of selection bias in those who are referred. Also as these were enrolled from secondary care, it is possible that more 'higher grade' of NAFLD may have been included compared to in the general population. We matched age and sex from a cohort of patients with full range of NAFLD severities to DILI participants in the discovery cohort.<br>HV were recruited through advertising to visitors in the Nottingham University Hospitals NHS Trust, NIHR Nottingham Biomedical Research Centre, so this was affected by the motivation of individuals to support research and local population composition.<br>Participants were offered reasonable travel expenses to attend study visits in excess of standard of care, but did not receive any inconvenience allowance or payment.
Ethics oversight	Yorkshire and the Humber - Leeds East Research Ethics Committee (Ref. 15/YH/0294); Biomedical Investigation Ethics Committee of Andalucia (Ref: AND-HEP-2015-01); Ethical Commission of Ludwig Maximilian University of Munich (Project 85-16); Bioethics Committee Iceland (Ref: 15-104-V1); Ethics Comission of Centro Académico Médico de Lisboa (Ref: 126/15) and National Data Protection Comission Portugal (Authorization 479/2016). For NAFLD and HV by East Midlands Nottingham 2 research ethics committee (Ref GM0102010).

Note that full information on the approval of the study protocol must also be provided in the manuscript.

Field-specific reporting

Please select the one below that is the best fit for your research. If you are not sure, read the appropriate sections before making your selection.

[x] Life sciences [ ] Behavioural & social sciences [ ] Ecological, evolutionary & environmental sciences

For a reference copy of the document with all sections, see nature.com/documents/nr-reporting-summary-flat.pdf

Life sciences study design

All studies must disclose on these points even when the disclosure is negative.

Sample size	Calculated based on sample availability, sample integrity and informed consent
Data exclusions	Missing patient samples were excluded from the study
Replication	We included confirmation and replication cohorts (Fig 1).
Randomization	There was no allocation of participants to groups as this is not a clinical trial - samples were from a patient registry
Blinding	All lab analysis was performed by scientists blinded to the sample type/cohort.

Reporting for specific materials, systems and methods

We require information from authors about some types of materials, experimental systems and methods used in many studies. Here, indicate whether each material, system or method listed is relevant to your study. If you are not sure if a list item applies to your research, read the appropriate section before selecting a response.

Materials & experimental systems

n/a	Involved in the study
x	Antibodies
x	Eukaryotic cell lines
x	Palaeontology and archaeology
x	Animals and other organisms
	x Clinical data
x	Dual use research of concern

Methods

n/a	Involved in the study
x	ChIP-seq
x	Flow cytometry
x	MRI-based neuroimaging

Clinical data

Policy information about clinical studies

All manuscripts should comply with the ICMJEguidelines for publication of clinical research and a completedCONSORT checklist must be included with all submissions.

Clinical trial registration	n/a - it is not a clinical trial
Study protocol	Pro-Euro-Dili Registry: A Collaborative Effort to Enhance the Understanding of Dili<br>Slim, M. et al.<br>Journal of Hepatology, Volume 64, Issue 2, S293 - S294
Data collection	Suspected DILI cases were consecutively recruited at secondary care centers across Europe between April 2016 and July 2021: UK, Spain, Germany, Iceland, Portugal.Healthy volunteers (HV) and patients with biopsy-proven chronic liver disease (nonalcoholic fatty liver disease: NAFLD) were enrolled as additional control groups. These controls were sex and age matched to the DILI cases and were recruited prospectively in parallel in dedicated research facility within secondary care setting in Nottingham, UK .
Outcomes	n/a registry cohort
